# Supplementary material for: Non-invasive electromechanical assessment during atrial fibrillation identifies underlying atrial myopathy alterations with early prognostic value
Source: Nat Commun. 2023 Aug 4;14:4613. doi: 10.1038/s41467-023-40196-y (PMC10403561; doi:10.1038/s41467-023-40196-y)
Supplement: Supplementary file 1 — Supplementary Information [file 41467_2023_40196_MOESM1_ESM.pdf]

Supplementary Material

Non-invasive electromechanical assessment during atrial fibrillation identifies underlying atrial myopathy alterations with early prognostic value

Supplementary Methods

In vivo electroanatomical mapping in pigs with persistent atrial fibrillation

Electroanatomical mapping procedures were performed under general anesthesia. All pigs were pre-medicated with a combination of intramuscular Ketamine (20 mg/kg) and Midazolam (0.5 mg/kg). Anesthesia was induced with an intravenous bolus of Fentanyl (0.010 mg/kg). After oral tracheal intubation animals were mechanically ventilated with intermittent positive pressure and anesthesia was maintained by a combination of Fentanyl (0.005 mg/kg/h i.v.) and Sevoflurane (2%). Vital signs were monitored during the procedure to control anesthesia status. The invasive electrophysiology study was performed using percutaneous venous access to reach the atrial chambers. Arterial femoral access was used for continuous monitoring of blood pressure. Intracardiac signals and blood pressure were continuously monitored using the LabSystem-Pro electrophysiology recording system (Boston Scientific, Lowell, MA). During the mapping procedure, ventricular pacing was adjusted between 40 and 60 beats per minute to minimize the number of ventricular artifacts on atrial signals while ensuring a mean blood pressure >70 mm Hg. Mapping procedures were guided with the Ensite Precision (Abbott, Princeton, NJ) using an internal reference decapolar catheter (Dynamic XT, Boston Scientific, Marlborough, MA) positioned into the superior vena cava. The distal electrode of the decapolar catheter was also used as unipolar reference. A PentaRay catheter (20 poles, Biosense Webster, Diamond Bar, CA) was introduced through a long steerable sheath (8.5 F Agilis NxT Steerable Introducer, 91 cm) to sequentially map different locations of the right atrium and coronary sinus. Eight-second-long unipolar signals were acquired at each location. Surface ECG signals were also simultaneously recorded in the Ensite Precision. Upon completion of the right atrial map, a transeptal puncture was performed to get access to the left atrium and further complete endocardial mapping in this chamber. Heparin (130 IU/kg) was administered to prevent thrombi formation after the transeptal puncture. Endocardial DF maps were generated using unipolar electrograms from each mapping site. Ventricular far-field was cancelled using principal component analysis and other advanced signal processing techniques, as reported elsewhere.[1]

Endocardial activation rates and surface expression on Lead II ECG

In pigs with long-standing persistent atrial fibrillation (>6 months in self-sustained persistent AF), simultaneous acquisition of lead II recordings during electroanatomical mapping enabled us to quantify surface ECG expression of underlying endocardial activations rates. Similar to limb leads I, and III, atrial deflections in lead II contain both right and left atrial activity. However, limb lead II was selected based on previous reported data indicating that this lead provides the highest prediction performance in patients undergoing pharmacological cardioversion.[2] Lead II recordings were digitally acquired throughout the mapping procedure using an electrophysiology recording system (LabSystem Pro, Boston Scientific). The average duration of such ECG recordings was 141±39 min that were segmented into 1416±389 windows of 6-second duration. After removing ventricular pacing artifacts (i.e., pacing spikes) and QRS-T complexes using Principal Component Analysis (PCA),[3] the resulting atrial activations were spectrally analyzed to obtain their dominant frequencies (DFs). Endocardial DF maps were generated using unipolar electrogram signals for each recording site after ventricular far-field removal using PCA and other advanced signal processing techniques, as reported elsewhere.[1] Afterwards, local DF values between percentiles 5th and 95th on lead II were detected on the endocardial DF map to quantify the endocardial surface comprising DF values detected on lead II. Overall, during the electroanatomical mapping procedure 70.9±11.8% of the electrically active atrial endocardial surface showed local DF values within the 5th-95th percentiles of lead II-derived atrial electrical activation rates. An example is shown in **Suppl. Fig. 2**.

Atrial echocardiography measurements

In pigs, left atrial dimensions were measured using transthoracic echocardiography (TTE, probe X5-1) to obtain a four-chamber view with the animal in left lateral decubitus position. Left atrial area was measured at end-systole, on the frame just prior to mitral valve opening by tracing the left atrium inner border, excluding the area under the mitral valve annulus, the inlet of the pulmonary veins and left atrial appendage, similar to recommended methodology in humans (**Suppl. Fig. 1**).[4] Atrial dimensions in pigs were indexed to animal weight as follows:

$$\text{Left atrial area index} = \frac{\text{left atrial}_{\text{area}}}{\frac{970}{10000} * \text{weight}^{0.633}} \left(\frac{\text{cm}^2}{\text{Kg}^{0.633}}\right) \qquad (1)$$

The animal weight limit in our crossbred Yucatan-large white pigs was set at 80 Kg aiming to have a more realistic fat-free mass after such weight.

Left ventricular ejection fraction was measured using the Teichholz formula and M-mode echocardiography data. The maximum minor axis of the left ventricle at end-diastole and end-systole was measured on the parasternal long-axis or short-axis view. Tissue Doppler imaging (TDI) sequences in pigs were obtained using transesophageal echocardiography (TEE) views focused on the posterior left atrial wall. In a subgroup of 7 pigs with healthy atria and 8 pigs with long-lasting persistent AF, TDI sequences were also obtained from TEE views focused on the left atrial free wall.

In patients, left atrial area was planimetered in the apical four chamber view. Optimal contours were obtained orthogonally around the long axis of the left atrium from good quality images while avoiding foreshortening. Left atrial area was measured at end-systole, on the frame just prior to mitral valve opening by tracing the left atrial inner border, excluding the area under the mitral valve annulus and the inlet of the pulmonary veins.[4] Three-dimensional atrial volumes were obtained from the apical view using a multibeat full-volume acquisition. The upper normal limit for left atrial volume was set at 34 ml/m2 for both genders, as reported elsewhere.[4] The anteroposterior diameter of the left atrium was measured in the parasternal long-axis view perpendicular to the aortic root long axis.

Right atrial areas were measured in the apical four-chamber view at end-systole, on the frame just prior to tricuspid valve opening, by tracing the right atrium blood-tissue interface, excluding the area under the tricuspid valve annulus.[4] Right atrial areas were indexed to indexed to body surface area. The upper normal limit for right atrial area was set at 11 cm^2/m2 for both genders, as reported elsewhere.[5]

Left atrial global longitudinal strain was calculated as the longitudinal strain obtained from a non-foreshortened apical four-chamber view.[6] The same echocardiographic view was used for the right atrial global longitudinal strain. Conventional atrial strain parameters include strain curves with the reservoir phase, the conduit phase and contraction phase. However, in AF the conduit phase will continue until the end of ventricular diastole. Therefore, in patients with AF only two phases can be used to assess atrial strain; one during the reservoir phase and a second one during conduit phase. However, in AF, the conduit phase has the same value as the reservoir phase, but with a negative sign. Therefore, we only analyzed the reservoir phase.

Fibrosis quantification and Real Time quantitative PCR analysis in atrial biopsies

For histological studies, myocardial biopsy samples were fixed in 4% formalin for at least 14 days and then transferred to 70% ethanol. Afterward, cross-sections of the myocardium were dehydrated, embedded in paraffin, and cut into 5-µm thick sections. Tissue slices were stained with Picro-Sirius Red and digitized using a NanoZoomer S360 Digital slide scanner (Hamamatsu, Japan) for analysis. A total of 10 randomly selected 40X insets per sample were analyzed (excluding endocardial, epicardial, and peri-vascular regions). Interstitial fibrosis was quantified using ImageJ with a modified version of a color deconvolution-based plugging.[7] Interstitial fibrosis proportion (red stained areas/total area) was measured in every inset, and a mean value was assigned to each sample.

Gene expression analysis was performed by RT-qPCR. Total RNA was isolated from myocardial frozen samples by using TRIzol Reagent (Ref – 15596026, Thermo Fisher Scientific, Massachusetts, USA) following the standard protocol. cDNA was synthesized from total RNA using the Applied Biosystems

High-Capacity cDNA Reverse Transcription kit (Ref – 4368814, Thermo Fisher Scientific, Massachusetts, USA). Quantitative PCR (RT-qPCR) was conducted with SYBR Green (Ref – 4367659, Thermo Fisher Scientific, Massachusetts, USA) where cDNA was amplified by using custom DNA primers (Suppl. Table 2) designed for every gen based on the pig genome (Table below). The GAPDH gene was used as housekeeping gene for gene expression normalization, and the $2^{-\Delta\Delta Ct}$ method was used for relative gene expression quantification.[8]

Immunohistochemistry and confocal microscopy

- Confocal microscopy of TUNEL staining: Frozen samples embedded in OCT were sectioned using a cryostat microtome (Leica CM1850). 10-µm thick cryosections were collected on microscope glass slices and fixed by adding 2% paraformaldehyde (PFA) for 10 min. Samples were then washed three times with PBS. The *in situ* cell death detection kit (Sigma-Aldrich) was used for the detection and quantification of apoptosis at the single-cell level according to manufacturer's instructions. Thereafter, samples were incubated with wheat germ agglutinin (1:400, Thermo Fisher Scientific) and DAPI (1:500, Thermo Fisher Scientific). Finally, the samples were mounted in VECTASHIELD mounting medium (Vector Laboratories). Fluorescence images of labeled tissue slices were acquired using a confocal microscope (Leica SP8X, 405 nm laser, white light laser, glycerol immersion objective: HC PL APO 20x) with suitable laser lines. Sensitive hybrid detectors were used to image fluorescence. TUNEL-positive cells were quantified in ImageJ platform (http://imagej.nih.gov/ij/) using an automated macro.

- Caspase 3 staining: Atrial and ventricular tissue samples were fixed in 4% formalin and then transferred to 70% ethanol. Samples were embedded in paraffin blocks and cut in 5µm sections. Blocks were cut onto slices and dried overnight at 37ºC and 2 hours at 65ºC. Slices were deparaffinized and hydrated. After antigen retrieval in Tris/EDTA buffer (pH 9), sections were incubated with Rabbit polyclonal Caspase 3 antibody (ASP175, Cell Signaling) for 45 min at room temperature followed by Envision HRP labelled polymer (K4003, Dako). DAB (3,3′-Diaminobenzidine) liquid (K3468, Dako) was used for the development of the signal (brown) followed by Hematoxylin for nuclear staining (blue). Stained sections were scanned and then 20X magnification images were used for visualization of left atrial and left ventricular samples. Images were analyzed with Fiji ImageJ software with a home-made script developed by the Microscopy Unit at CNIC.

Proteomic analysis

Atrial biopsies protein extracts were obtained by tissue homogenization with ceramic beads (MagNa Lyser Green Beads, Roche, Germany) in CS buffer (Pipes pH6.8, MgCl2, NaCl, EDTA, sucrose, SDS, sodium orthovanadate; Biochain Institute, Inc. #K3013010-5) freshly supplemented with protease and phosphatase inhibitors. Extracted proteins (~200 µg) from atrial biopsies were subjected to in-filter reduction and alkylation using iodoacetamide followed by trypsin digestion (Nanosep Centrifugal Devices with Omega Membrane-10K, PALL), and the resulting peptides were TMT-labeled following manufacturer's instructions. Labeled peptides were subjected to LC-MS analysis using a Proxeon Easy nano-flow HPLC system (Thermo Fisher Scientific, Bremen, Germany) coupled via a nanoelectrospray ion source (Thermo Fisher Scientific) to an Orbitrap Fusion mass spectrometer (Thermo Fisher) using with a 2-cm trap column and a 50-cm analytical column (75 µm I.D, 2 µm particle size, Acclaim PepMap RSLC, 100 C18; Thermo Fisher Scientific) in a continuous acetonitrile gradient consisting of 0-30% A for 60 min, 50-90% B for 3 min (A= 0.1% formic acid; B= 100% acetonitrile, 0.1% formic acid) at a flow rate of 200 nL/min. Mass spectra were acquired in a data-dependent manner, with an automatic switch between MS and MS/MS using a top-speed method and dynamic exclusion. MS spectra were collected in the Orbitrap analyzer using a mass range of 400–1500 m/z at 60,000 resolution. HCD fragmentation was performed at 33 eV of normalized collision energy and MS/MS spectra were analyzed at 30,000 resolution in the Orbitrap.

Protein identification was performed using the SEQUEST HT algorithm integrated in Proteome Discoverer 2.5 (Thermo Scientific). MS/MS scans were searched against a pig reference proteome database (human_pig_202105_pro-sw-tr.target-decoy.fasta), (296316 sequences in total). For database

searching, parameters were selected as follows: trypsin digestion with 2 maximum missed cleavage sites, precursor mass tolerance of 2 Da, fragment mass tolerance of 0.03 Da. Methionine oxidation (+15.994915 Da) and asparagine and glutamine deamidation (+0.984016 Da) were set as variable modifications, while cysteine carbamidomethylation (+57.021464 Da) and TMT labeling (+229.162932 Da) at peptide N-terminal end and Lys were considered as fixed modifications. False discovery rates (FDR) of peptide identifications were calculated using the refined method with an additional filter for precursor mass tolerance of 10 ppm[9]. 1%FDR was used as criterion for peptide identification.

Quantitative information from TMT reporter intensities was integrated from the spectrum level to the peptide level, and then to the protein level based on the WSPP model [10, 11] using the GIA integration algorithm. Relative protein abundances were expressed as standardized log2-ratios (*Zq*).

Proteomics Data Source: Triplicates for biopsies D and B were labeled with reagents 126, 127N, and 127C in experiments TMT1 and TMT2, respectively. Triplicates of biopsies A and F were labeled with reagents 128N, 128C, and 129N in experiments TMT1 and TMT2, respectively. The mixture of all the replicates was labeled with reagent 131, and was used as internal standard in the two TMT experiments.

Western blotting

Protein extracts were obtained by disruption in a Tissue Lyser (Qiagen) for 15 min at 1/50 s., followed by homogenization in Radioimmunoprecipitation assay (RIPA) sample buffer (Sigma-Aldrich) containing protease and phosphatase inhibitors (Roche). Total protein concentration was quantified with Pierce™ BCA protein assay kit (Thermo Fisher Scientific). Proteins (10 to 15 µg) were separated with 6%, 7.5%, 10% or 15% (according to the molecular weight of the targeted proteins) sodium dodecyl sulfate-polyacrylamide gel electrophoresis (SDS-PAGE) and transferred to 0.45 µm polyvinylidene difluoride (PVDF) Immobilon®-P transfer membrane (Merck, Millipore) for Ryanodine Receptor 2 (RyR2) and pSer2814 RyR2 at 50 mA and 4º C during 22 hours in a tank transfer system (Bio-Rad), or 0.2 µm nitrocellulose (Bio-Rad) membranes at 2.5A 10V during 10 min in TransBlot® Turbo™ System (Bio-Rad) for the other proteins. Total protein determination was performed with Pierce™ Reversible Protein Stain Kit for PVDF or nitrocellulose membranes (Thermo Fisher Scientific) in Gel Doc XR^+ system (Bio-Rad). Blocking (1 h at room temperature.) and antibody (Ab) incubations was performed in 5% bovine serum albumin (BSA) in Tris buffered saline (TBS) Tween 20© 0.2%. Primary Ab against cardiac muscle alpha-actin (ACTC1), Bcl-2-associated X protein (BAX), myosin-binding protein C cardiac (MYBPC3), sodium/calcium exchanger (NCX), phospholamban (PLN), pThr17 PLN, sarco-endoplasmic reticulum calcium ATPase (SERCA2), RyR2, and pSer2814 RyR2, were incubated overnight under agitation at 4º C. Detection was performed after HRP-conjugated anti-mouse or anti-rabbit incubation for 1h at room temperature by Immobilon® Western Chemiluminescent HRP Substrates (Merck, Millipore). Concentrations and references for all the Ab used are shown in Suppl. Table 3.

Specific protein bands were visualized in ImageQuant LAS 4000 mini Biomolecular Imager (GE Healthcare). Quantitative densitometry was performed using Fiji (ImageJ) software.

Computational modeling

Human atrial action potential and its corresponding calcium transients were simulated using the mathematical model described by Skibsbye *et al.*[12] AF remodeling scenarios were simulated by introducing pertinent changes in model parameters. These changes were based on previous series reporting: cell dilation (+10%), I_{CaL} (–55%), I_{to} (–62%), I_{Kur} (–38%), I_{K1} (+68%), INa (–18%), IKs (+145%). In addition to ion currents remodeling (i.e., electrical remodeling), the phospho-phospholamban parameter was adjusted according to our experimental results (-82%) to simulate alterations in sarco-endoplasmic reticulum ATPase2 (SERCA2) activity and abnormal calcium handling. We specifically tested 3 simulation scenarios: i) AF without remodeling (based on the model by Skibsbye *et al.*[12]*; ii)* AF with electrical remodeling (based on ion current changes described by Skibsbye *et al.*[12]*)*, and *iii)* AF with electrical remodeling and additional calcium handling changes

associated with the -82% decrease in Thr17-pPLN/Total-PL documented in the experimental data **(Suppl. Fig. 8)**.

The mathematical model was implemented in Matlab (The MathWorks) and simulation results were obtained by numerically integrating the model equations with a stiff ordinary differential equation solver method (ode15s). The simulations were performed using random irregular pacing rates from 3 to 7 Hz for 6 seconds after a steady state of 4-second pacing at 7 Hz (total duration 10 seconds).

Di-4-ANEQ(F)PTEA and Fura-2AM dye loading protocol

Excised hearts were submerged in cold (4 ºC) Tyrode's solution, cleaned and then cannulized through the aorta and connected to a constant-flow Langendorff-perfusion apparatus. After atrial trans-septal puncture, all the vein orifices were sealed except the inferior vena cava, which was connected to an open-end cannula to control the intra-atrial pressure. The intracavitary pressure was increased to a level of 6-8 cm H_2O, resembling the diastolic left atrial pressure and maintained throughout the experiment.(*3,19*) Hearts were perfused with oxygenated (95% O_2, 5% CO_2) Tyrode's solution (composition in mM: NaCl 130, $NaHCO_3$ 24, NaH_2PO_4 1.2, $MgCl_2$ 1, Glucose 5.6, KCl 4, $CaCl_2$ 1.8 and albumin 0.04 g/L) at a circulating flow rate of 200-240 mL/min. Ionic pH of the perfusate (7.4), oxygenator status and temperature (36.5-37.5 ºC) were monitored throughout the experiment. After an initial 25 min of perfusion following cannulation, the heart was submerged inside a tank filled with saline, maintained at 37ºC. After 5-10 min inside the tank, the heart underwent a recirculating perfusion of 1 L of perfusate containing 1 mM probenecid (50027; Biotium Inc., Hayward, CA, USA) and 3 µM fura-2AM (1051B, stock 1 mg/mL in DMSO; Ion Biosciences LLC, San Marcos, TX, USA). After 20 min, 600 µL of di-4-ANEQ(F)PTEA stock dye solution (10 mg dissolved in 3 mL of pure ethanol; University of Connecticut School of Medicine, Farmington, CT, USA) diluted in 10 mL of Tyrode's solution was delivered slowly over a 1-min period a short distance upstream from the aortic cannula for coronary perfusion. After 10 min, and before mapping, the recirculating perfusate was replaced with fresh Tyrode's solution without probenecid.

Twelve-lead ECG digitation

Printed 12-lead ECG recordings before flecainide administration were scanned and converted to an uncompressed maximal quality image in jpg format. Then, we used a customized tool written in Matlab to digitize and analyze all 12 ECG leads. The scanned image was loaded and accurately rotated in case it was not properly scanned in a completely straight fashion. Then, several lines could be manually adjusted to mark the horizontal and vertical separations between every 2 adjacent leads. The latter enabled the operator to separate the image sections that contained all the 12 ECG leads and the 10-second rhythm lead II, to further display them on a new figure. The image section containing an ECG lead was converted to grayscale, and a threshold was automatically determined to binarize the image (black and white). Then, continuous black objects with fewer than a specific number of pixels were removed since they were likely parts of the underlying grid. This specific number of pixels was automatically determined by the tool according to the resolution and size of the image. After checking that the final digitized signal faithfully followed the ECG trace on the scanned image, the process moved to the next step. This step was repeated 13 times, once for each ECG lead plus the 10-second lead II strip.

QRS-T removal and spectral analysis of ECG-derived atrial electrical activity

Before QRS-T removal, the software tool enabled the operator to: 1) remove linear trends in the digital ECG signal; 2) perform additional median filtering, if needed; 3) perform notch filtering to remove the 50/60 Hz electrical noise from the network if present; and 4) perform high pass filtering to remove low frequency components (e.g., baseline wander). Although, the more filtering the less noise, this filtering process may also introduce distortion on the signal. Therefore, one or more of these processes were performed only if they were strictly necessary. The latter was not often the case due to the prospective inclusion criteria and high-quality of the ECGs acquired for this study. Afterward, the operator selected and accurately aligned the QRS-T complexes in the extracted signal by detecting fiducial times through

a customizable band-pass filtered version of the original signal. The maximal QR and RT interval lengths were also adjusted by the operator. Once the operator was satisfied with the lengths and alignment of the detected QRS-T complexes, an estimation of the ventricular QRS-T complex was subtracted from the ECG signals. This estimation was based on PCA, as described elsewhere.[1, 3] More specifically, the PCA method was applied to the time windows set in the previous step, which comprised from the beginning of the Q-wave (or the spike of the stimulus if ventricular pacing was present, as in ECG tracings from the animal model) to the end of the T-wave. The same window size was used for all the QRS-T complexes within an ECG signal. In the PCA method, the QRS-T complexes from the ECG tracings are considered as realizations from stochastic processes for which a covariance matrix is derived. Then, the eigenvectors and eigenvalues of the covariance matrix are calculated, with the eigenvectors placed in one matrix E and the eigenvalues placed in a diagonal matrix D. From these two matrices, a whitening matrix (a matrix that transforms the original observation matrix X into a matrix of whitened principal components) and a dewhitening matrix (a matrix which undoes the whitening process) are derived:

Whitening Matrix*:* $W = D^{-1/2}E^T$ (2)

Dewhitening Matrix*:* $W^{-1} = ED^{1/2}$ (3)

The principal components can then be obtained from the observation matrix and whitening matrix by multiplication:

Principal Components: $P = WX$ (4)

These principal components were combined using their associated weights in the dewhitening matrix to create an individualized template for each QRS-T complex in the ECG signal. Since the first principal components are assumed to express the ventricular depolarizing and repolarizing activity because they express most of the variance from the original observations, QRS-T templates were created by combining the first 1-2 principal components, using their associated mixing variables from the dewhitening matrix W^{-1}. These QRS-T templates (red tracings in **Suppl. Fig. 11**) were then subtracted from the original ECG signal (black tracing in **Suppl. Fig. 11**) to obtain the atrial signal (blue tracing, bottom row in **Suppl. Fig. 11**).

Then, the DF of the atrial ECG signal was calculated as the frequency with the highest peak in the power spectral density (PSD) within the 3-15 Hz interval. This provided a robust and rapid calculation of the reciprocal average electrical activation intervals:

$$\text{Average cycle length (ms)} = \frac{1000}{\text{DF (Hz)}} \qquad (5)$$

PSD was estimated by a Welch's periodogram of the signal, which was previously multiplied by a Hamming window and zero-padded to the next higher power of 2. One investigator (D.E.V) blinded to clinical outcomes reviewed all ECG-derived DF values to detect potential harmonic peaks. Atrial signal visualization was less reliable in left precordial leads. Therefore, DF values from atrial signals on these leads (from V3 to V6) were not used for univariate analysis.

Extraction and processing of Tissue Doppler Imaging signals

Data from tissue Doppler imaging sequences were exported in DICOM format and further processed to obtain raw TDI signals from the entire window inside the image sector angle. TDI signals were analyzed using an additional customized tool implemented in Matlab. The data exported from the echocardiography apparatus included: i) 6-second 2D echocardiography movies, ii) TDI data from a specific region within the 2D images and iii) simultaneous lead II ECG signals. After loading the data into a custom-made software tool, the operator could visualize both the echocardiography movie and TDI data on a superimposed layer. The tool also enabled the possibility to perform a smoothing gaussian space filtering of the echocardiography images (optional). However, this capability was rarely used in this study due to the high quality of the echocardiography movies acquired. The next step consisted on

manually demarcating the contour of the atrial wall on an average 2D echocardiography image. Using such a contour as an initial reference, an automatic algorithm tracked the atrial wall motion throughout the cardiac cycles within the acquisition interval. More specifically, 3 areas were demarcated with high (region A, default value: ≥75% of the maximum echogenicity), medium (region B, default value: ≥60% of the maximum echogenicity) and low echogenicity (region C, default value: ≥30% of the maximum echogenicity). Automatic but customizable morphological operations (erosion, closing, and removal of small disconnected groups of pixels) were performed to generate consistent A, B and C regions throughout the whole echocardiography movie. These A, B and C regions could be displayed over the echocardiography movie as dynamic red, green and blue masks, respectively. All local TDI signals from every pixel within regions A, B and C were averaged to generate 3 TDI signals with improved quality to work with. Overall, region C provided good and reliable TDI signals in both pigs and patient studies.

Ventricular motion artifacts were also present in some TDI signals. Therefore, the next step included subtraction of mechanical artifacts produced by ventricular contractions from the averaged TDI signals. For this purpose, the tool implemented 2 different methodologies: PCA (as in surface ECG tracings) and Empirical Mode Decomposition. Similar to QRS-T removal, PCA-based subtraction enabled the operator to: 1) remove linear trends in the TDI signal; 2) perform median filtering; 3) perform notch filtering to remove the 50/60 Hz electrical noise from the network, if present; and 4) perform high pass filtering to remove low frequency components. One or more of these processes were only performed if they were strictly needed in order to avoid potential distortion. Then, the fiducial times detected on the simultaneous ECG lead II signal were used to select and align the consequent mechanical artifacts on TDI signals produced by the ventricular contraction. Subsequently, a PCA-based estimation of the ventricular mechanical artifact on TDI signals was subtracted from the signals. In the example shown in **Suppl. Fig. 12A, B,** using 1 component to estimate the ventricular mechanical artifact, the resulting signal (blue thick tracing in panel B) should have displayed the underlying atrial mechanical activity. However, although the PCA methodology was highly effective on ECG signals, its performance with DTI signals was somehow disappointing. In addition, this example shows a spectrum with several peaks of similar height in which DF selection may be arbitrary. In the same sample TDI trace, the results were even worse using 2 components to estimate the ventricular mechanical artifact. As shown in **Suppl. Fig. 12C** the unmasked alleged atrial mechanical activity was almost negligible in the intervals concurrent with ventricular artifacts. In addition, the frequency of the highest peak in the spectrum was 3.1 Hz, which clearly not matched the atrial mechanical activity on the intervals free from ventricular artifact. Another limitation for PCA-based subtraction of ventricular motion artifacts on TDI signals is that irregular activation of the ventricles during AF results in different levels of ventricular contraction depending on the previous diastolic interval, modulating the morphology of the ventricular artifacts on TDI signals. Thus, ventricular mechanical artifacts on TDI signals may not display a reproducible morphology, unlike electrical QRS-T complexes that mostly display a reproducible morphology during the entire ECG segment. These and other additional factors may undermine the effectiveness of the PCA methodology to extract the underlying atrial mechanical activity on TDI signal.

In this context we implemented and tested other potential methodologies to extract the atrial mechanical activity hidden under the mechanical artifacts produced by ventricular contraction. After considerable testing, we decided to use the Empirical Mode Decomposition algorithm.[13] This method was developed from the assumption that any non-stationary and non-linear signal consists of different simple intrinsic modes of oscillation. The essence of the method is to empirically identify these intrinsic oscillatory modes by their characteristic time scales in the data, and then decompose the data accordingly. Thus, it adaptively and locally decomposes any non-stationary signal in a sum of Intrinsic Mode Functions (IMFs) that represent zero-mean, amplitude- and frequency-modulated components. The empirical nature of Empirical Mode Decomposition offers the advantage over other signal decomposition techniques of not being constrained by conditions, which often only apply approximately. Thus, the Empirical Mode Decomposition represents a fully data-driven, unsupervised signal decomposition and does not need any a priori defined basis system; i.e., it is really based on the dataset and does not have an assumption about the data (that is why it is called Empirical). Empirical Mode Decomposition also satisfies the perfect reconstruction property, i.e. superimposing all extracted IMFs together with the

residual slow trend reconstructs the original signal without information loss or distortion.[14] The Custom-made software for processing raw TDI signals can be provided upon reasonable request.

Suppl. Fig. 13A shows an example of a synthetic signal obtained as the sum of a 7 Hz sinusoid (roughly simulating a 7 Hz atrial mechanical activity), a sinusoid with a quadratically increasing instantaneous frequency from 0.5 Hz to 2 Hz (roughly simulating a ventricular motion artifact of varying frequency), and a decreasing linear trend. **Suppl. Fig. 13B** shows a similar signal, but in this example, the sinusoid with a quadratically increasing instantaneous frequency from 0.5 Hz to 2 Hz, also displays decreasing amplitude (surrogate of ventricular motion of varying frequency and amplitude). In both examples shown in **Suppl. Fig. 13A, B**, Empirical Mode Decomposition successfully decomposed this signal into its underlying components with a negligible error. **Suppl. Fig. 14** shows an example of the signal in **Suppl. Fig. 13B** with and additional quadratic underlying baseline. In this scenario, Empirical Mode Decomposition fused together the linear and quadratic trends into a single IMF, but importantly, it successfully recovered the other 2 components with minimal error, including the 7 Hz sinusoid. **Suppl. Fig. 15** shows Empirical Mode Decomposition performance when non-negligible additive white gaussian noise was added to the signal showed in **Suppl. Fig. 14** (signal-to-noise ratio: 20 dB). In this challenging scenario, the algorithm decomposed the noisy signal into 10 components. The first 5 were exclusively high-frequency noise. IMF6 together with IMF7 mostly constituted the 7 Hz sinusoid. IMF8 plus IMF9 approximately yielded the sinusoid with a quadratically increasing instantaneous frequency from 0.5 Hz to 2 Hz and decreasing amplitude. Finally, IMF10 was the combination of the linear and quadratic trends. This example demonstrates the need for combining some of the extracted IMFs to create meaningful signals in a real-world scenario.

Suppl. Fig. 16A-B shows an example of the Empirical Mode Decomposition method using a real TDI signal from a patient with AF (same sample case as in **Suppl. Fig. 11, 12**). TDI signals were obtained from the left atrial wall using an apical 4-chamber view. The raw TDI signal was automatically decomposed into its IMFs. Then, the operator manually selected an interval displaying clear atrial motion, preferably without or with minimal ventricular artifact (in this example, an interval around t=5 second was selected). With this information, the tool automatically detected the partial combination of IMFs that best correlated with the original signal in the operator-guided selected interval. The operator could optionally remove or include additional IMFs into the estimated atrial motion signal. In this sample case, the single IMF with the highest correlation was IMF 5 (correlation: 0.78). If IMFs 4 and 6 were added, the correlation increased to 0.94 (the maximum correlation is 1), resulting in the blue signal. The residual signal (potentially the ventricular mechanical artifact) is shown in red. In this case, the spectrum of the atrial mechanical activity showed a clear peak at 5.9 Hz which was consistent with the simultaneous atrial electrical DF (6 Hz, see **Suppl. Fig. 11**), meaning that electromechanical uncoupling was negligible in this patient at that stage. Additional analysis including more IMFs components below 4 and above 6 did not change spectral analysis and the DF value to obtain mechanical activation rates (**Suppl. Fig. 16C**).

The performance of the Empirical Mode Decomposition method in pigs was also very reliable. The pig model had the advantage of enabling us to adjust the ventricular pacing rate to decrease ventricular motion artifacts. A sample case of Empirical Mode Decomposition performance is shown in **Suppl. Fig. 17**. We also tested Empirical Mode Decomposition performance using programmed atrial stimulation in specific subset of animals (N=16) with healthy atria undergoing programmed atrial stimulation and AF induction after burst pacing at 10 Hz (**Fig. 1C, E in the main text**).

After applying the Empirical Mode Decomposition method described above to TDI signals, the mean amplitude excursion of the resulting TDI signals was also calculated to assess any potential association between this parameter and the primary outcome of pharmacological cardioversion. For each TDI signal we quantified the amplitude excursion of both positive and negative deflections and the average value of such excursions (mean ΔTDI). Interestingly, mean ΔTDI in both right and left atria did not differ significantly between patients with and without successful pharmacological cardioversion (**Suppl. Fig. 9**).

Spectral resolution and frequency threshold for detecting electromechanical dissociation

The same spectral estimation method described above (Welch's periodogram of the signal previously multiplied by a Hamming window and zero-padded to the next higher power of 2) was used to calculate DF in both lead II atrial ECG and TDI signals. Both signals were acquired with a sampling rate of at least 200 Hz. To determine the minimal difference in DF values to consider that EMD is actually present, we used the concept of 'spectral resolution'. To understand such a concept, there are several points related to Signal Theory to consider: i) a sinusoidal signal of infinite duration is the purest signal in the frequency domain and it would be ideally displayed as a spectral line (all its power is concentrated at a single frequency); ii) a sinusoid of finite duration is displayed in the frequency domain not as a spectral line, but as a large spectral lobe with some width and some secondary lobes of much lower amplitude, which is due, in part, to a 'widowing effect' (the spectrum we actually see is the convolution of a spectral line and the spectrum of the function used for windowing that generates a finite duration signal); iii) the sampling rate used by the echocardiography apparatus for digitizing the signal (>200 Hz) and the power spectral density estimation method (Welch's periodogram) also influence the accuracy in the determination of the exact frequency of an specific peak in the spectrum.

All these factors make that two sinusoidal signals with a small frequency separation (Δf) are not discernable in the frequency spectrum, since they would be displayed as a single fused spectral lobe. However, there is a minimal Δf above which two distinct spectral lobes start to be discernible. Such a minimal Δf is called 'spectral resolution' and depends on all the points considered above. We performed some simulations using the worst-case scenario (minimal sampling rate, 200 Hz) and determined that the spectral resolution of the spectral estimation method used in this study was 0.32 Hz (see **Suppl. Fig. 18**). That means that we cannot be sure that spectral peaks separated <0.32 Hz are displaying truly different frequencies. For this reason, we established the frequency threshold for detecting electromechanical dissociation at 0.32 Hz. Please note that any modification in the spectral estimation method that could be performed to improve spectral resolution is limited by the sampling rate provided by the echocardiography apparatus and, in addition, would be at the cost of worsening other features (for example, amplifying the secondary lobes consequence of the finite duration of the analyzed signals, which could confound DF determination). The method we used here provided a good balance between spectral resolution and minimizing other potential confounding factors in DF determination.

Supplementary references

1. Quintanilla JG*, et al.* Instantaneous Amplitude and Frequency Modulations Detect the Footprint of Rotational Activity and Reveal Stable Driver Regions as Targets for Persistent Atrial Fibrillation Ablation. *Circ Res* **125**, 609-627 (2019).

2. Zeemering S*, et al.* The electrocardiogram as a predictor of successful pharmacological cardioversion and progression of atrial fibrillation. *Europace* **20**, e96-e104 (2018).

3. Filgueiras-Rama D*, et al.* Long-term frequency gradients during persistent atrial fibrillation in sheep are associated with stable sources in the left atrium. *Circ Arrhythm Electrophysiol* **5**, 1160-1167 (2012).

4. Lang RM*, et al.* Recommendations for cardiac chamber quantification by echocardiography in adults: an update from the American Society of Echocardiography and the European Association of Cardiovascular Imaging. *European heart journal cardiovascular Imaging* **16**, 233-270 (2015).

5. Zaidi A*, et al.* Echocardiographic assessment of the right heart in adults: a practical guideline from the British Society of Echocardiography. *Echo Res Pract* **7**, G19-G41 (2020).

6. Badano LP*, et al.* Standardization of left atrial, right ventricular, and right atrial deformation imaging using two-dimensional speckle tracking echocardiography: a consensus document of the EACVI/ASE/Industry Task Force to standardize deformation imaging. *European heart journal cardiovascular Imaging* **19**, 591-600 (2018).

7. Hadi AM*, et al.* Rapid quantification of myocardial fibrosis: a new macro-based automated analysis. *Cell Oncol (Dordr)* **34**, 343-354 (2011).

8. Livak KJ, Schmittgen TD. Analysis of relative gene expression data using real-time quantitative PCR and the 2(-Delta Delta C(T)) Method. *Methods* **25**, 402-408 (2001).

9. Bonzon-Kulichenko E, Garcia-Marques F, Trevisan-Herraz M, Vazquez J. Revisiting peptide identification by high-accuracy mass spectrometry: problems associated with the use of narrow mass precursor windows. *J Proteome Res* **14**, 700-710 (2015).

10. Navarro P*, et al.* General statistical framework for quantitative proteomics by stable isotope labeling. *J Proteome Res* **13**, 1234-1247 (2014).

11. Trevisan-Herraz M*, et al.* SanXoT: a modular and versatile package for the quantitative analysis of high-throughput proteomics experiments. *Bioinformatics* **35**, 1594-1596 (2019).

12. Skibsbye L*, et al.* Refractoriness in human atria: Time and voltage dependence of sodium channel availability. *J Mol Cell Cardiol* **101**, 26-34 (2016).

13. Huang NE*, et al.* The empirical mode decomposition and the Hilbert spectrum for nonlinear and non-stationary time series analysis. *Proc R Soc Lond A* **454**, 903-995 (1998).

14. Zeiler A, Faltermeier R, Keck IR, Tomé AM, Puntonet CG, Lang EW. Empirical Mode Decomposition - an introduction. In: *The 2010 International Joint Conference on Neural Networks (IJCNN)*. IEEE (2010).

Supplementary Figures

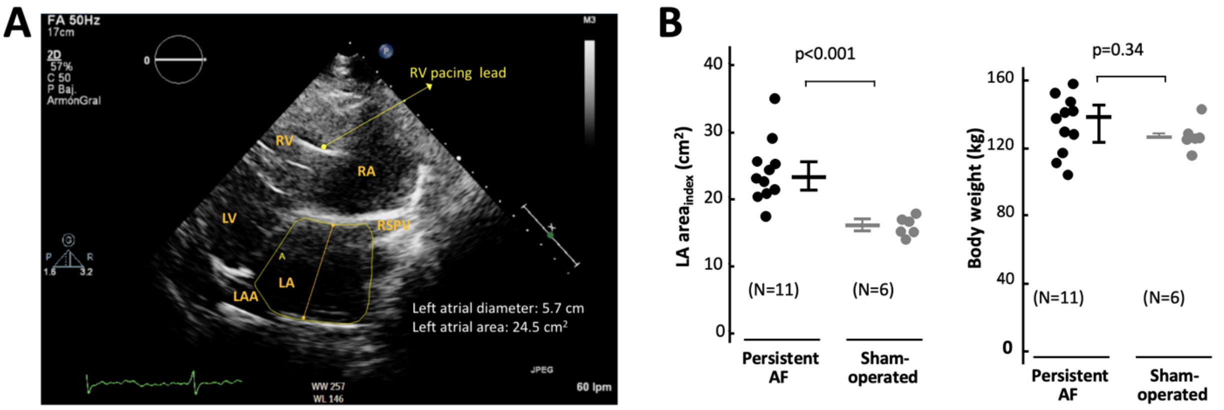

Suppl. Fig. 1. Echocardiography assessment of atrial dimensions in the pig model. A, Sample echocardiographic assessment of left atrial dimensions in the pig model. **B,** Comparisons of left atrial areas (indexed to body weight) and body weights between animals with long-lasting persistent atrial fibrillation (AF) and sham-operated controls. LA: left atrium. LAA: left atrial appendage. LV: left ventricle. RA: right atrium. RSPV: right superior pulmonary vein. RV: right ventricle. Box-plots show median and interquartile range. Two-sided unpaired Student's *t* tests were used to assess differences in panel B. Source data are provided as a Source Data file.

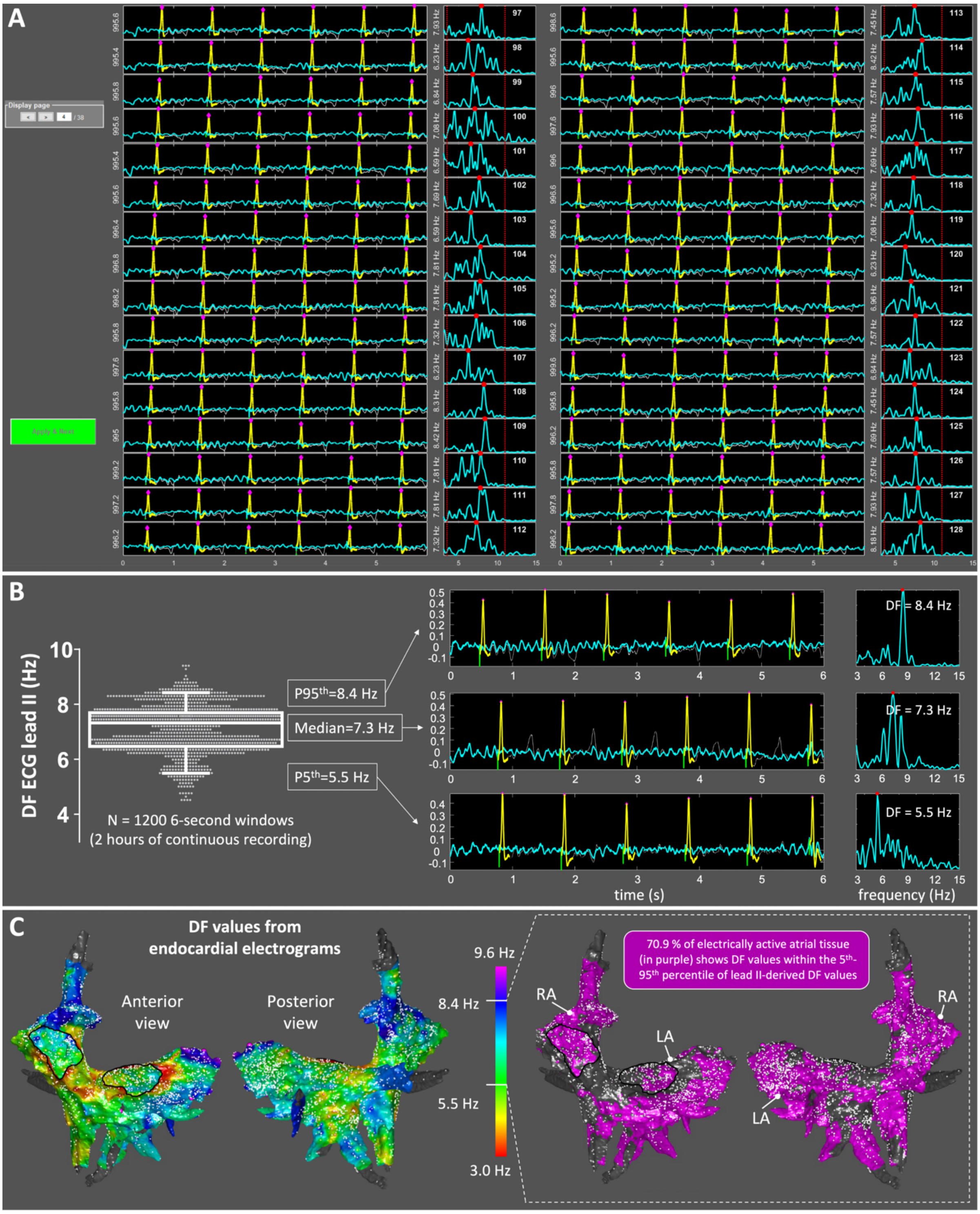

Suppl. Fig. 2. Quantification of the electrically active atrial/venous endocardial tissue within the range covered by dominant frequency values from lead II recordings. A, Example of the custom Matlab tool to obtain dominant frequency (DF) values from lead II ECG segments in a sample pig during the electroanatomical mapping procedure (2 hours, 1200 6-second ECG segments). **B,** Global distribution of the DFs measured in lead II during the electroanatomical mapping procedure in this pig. Right panels show specific examples of ECG segments with DFs values corresponding to percentiles 5th, 50th (median) and 95th of the global distribution. Ventricular pacing artifacts (green spikes) and QRS complexes (yellow) were automatically detected. The extracted atrial signal after Principal Component analysis is highlighted in cyan. **C,** Left, unipolar DF map of the left and right atria. Right, in this case 70.9% of the electrically active atrial/venous endocardial surface showed local DF values within the 5th-

95th percentile range (5.5-8.4 Hz, respectively) of lead II-derived atrial activation rates obtained from continuous ECG tracings during electroanatomical mapping. LA: left atrium. RA: right atrium.

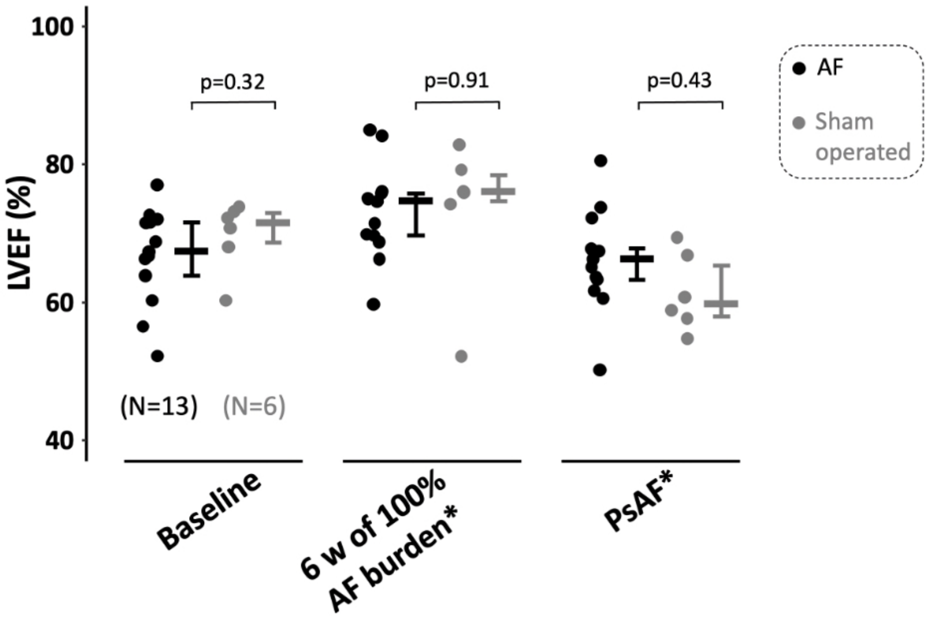

Suppl. Fig. 3. Quantification and comparisons of left ventricular ejection fraction over the follow-up of animals with atrial fibrillation and sham-operated controls. *indicates equivalent time-points of the follow-up in sham-operated controls. LVEF: left ventricular ejection fraction. PsAF: persistent atrial fibrillation. W: week. Box-plots show median and interquartile range. Two-sided unpaired Student's *t* tests were used to assess statistical significance. Source data are provided as a Source Data file.

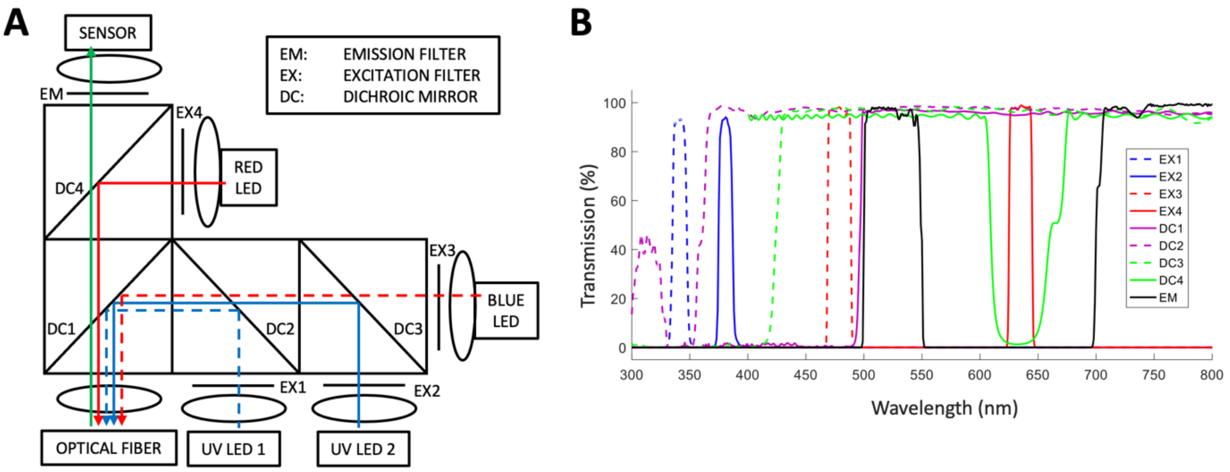

Suppl. Fig. 4. Optical mapping system for measuring voltage and calcium transients in contracting tissue through a single optical fiber. A, Schematic of the optical mapping system and light paths. The blue and red arrows (solid and dashed) show paths taken by the excitation lights; the green arrow shows the path taken by the emission light from both the voltage and calcium dyes. **B,** Transmission curves of all the excitation (EX), emission (EM) and dichroic (DC) filters shown in (A). LED: light-emitting diode.

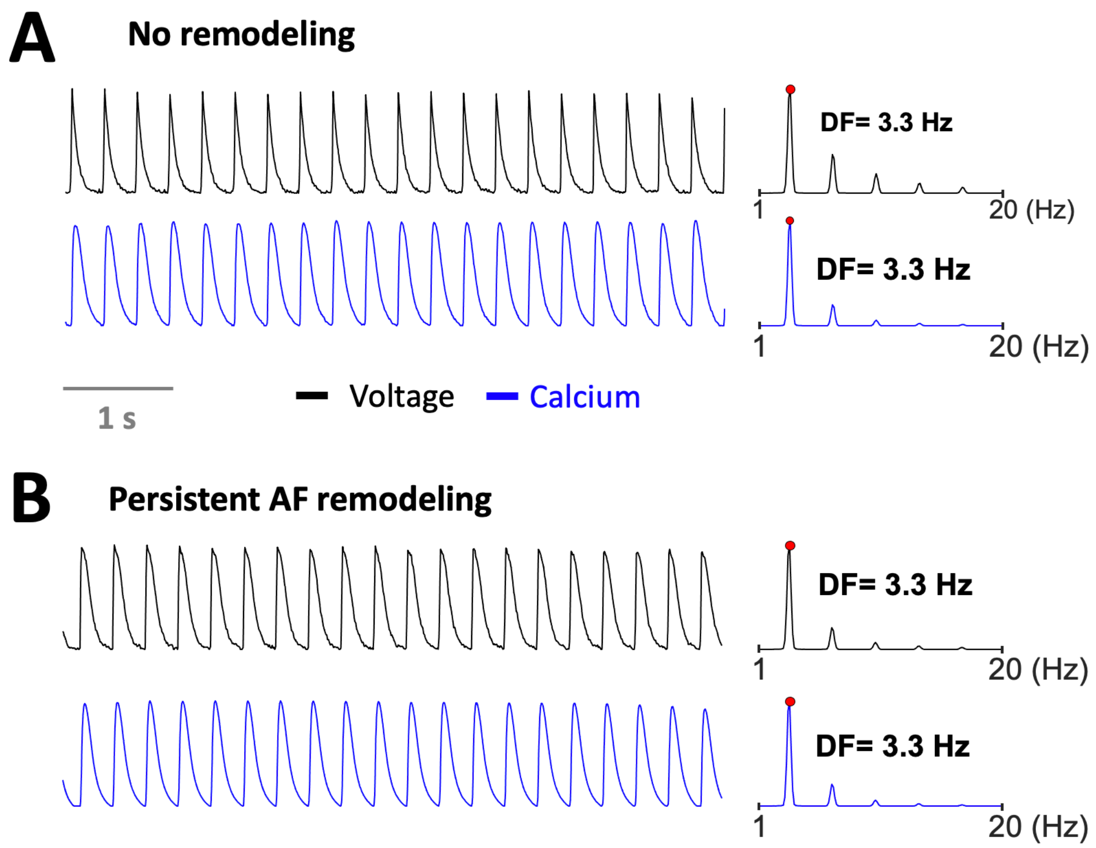

Suppl. Fig. 5. Optical mapping signals of simultaneous transmembrane voltage and intracellular free calcium during programmed atrial stimulation in isolated Langendorff-perfused pig hearts. Sample transmembrane voltage and intracellular calcium transients during atrial pacing at 300 ms in a control heart (A) and in a heart with atrial fibrillation (AF)-related remodeling (B). The signals shown are the ratio of the numerator and denominator for voltage and calcium to eliminate motion artifacts.

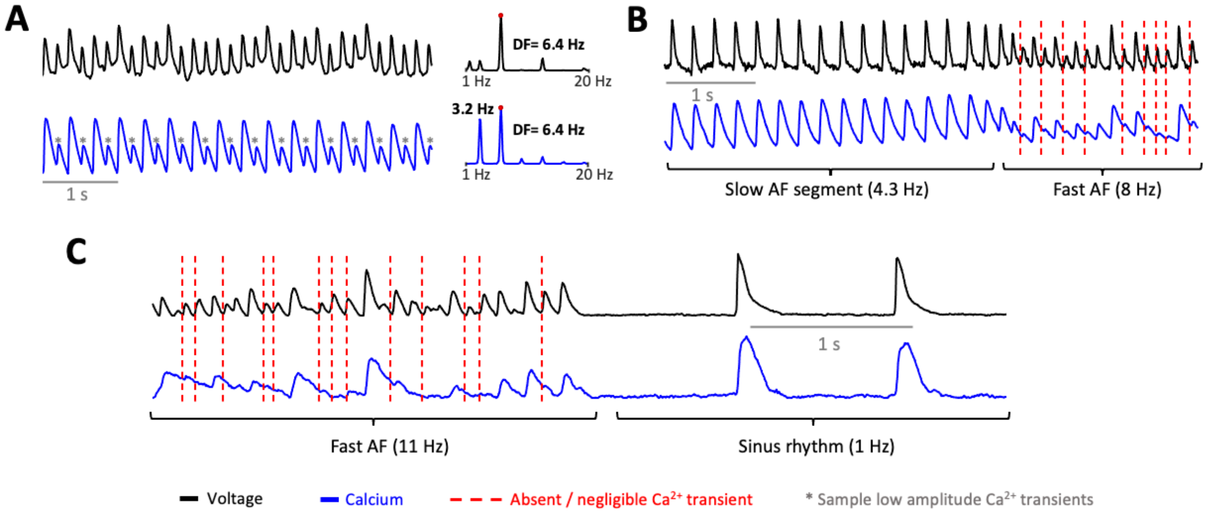

Suppl. Fig. 6. Sample optical mapping signals of simultaneous transmembrane voltage and intracellular free calcium during atrial fibrillation in isolated heart preparations. A, Sample calcium transient alternans during an atrial fibrillation (AF) episode at 6.4 Hz. **B,** Transmembrane voltage and intracellular free calcium signals during 2 AF segments with different activation frequencies: *(i)* slow (at 4.3 Hz) without voltage and calcium dissociation, and *(ii)* fast (at 8 Hz) with overt voltage and calcium dissociation. **C,** Transmembrane voltage and intracellular free calcium signals during an AF segment at high activation frequencies (11 Hz) and overt voltage and calcium dissociation, and a second segment after spontaneous cardioversion and sinus rhythm where voltage and calcium signals are no dissociated. The signals shown are the ratio of the numerator and denominator for voltage and calcium to eliminate motion artifacts.

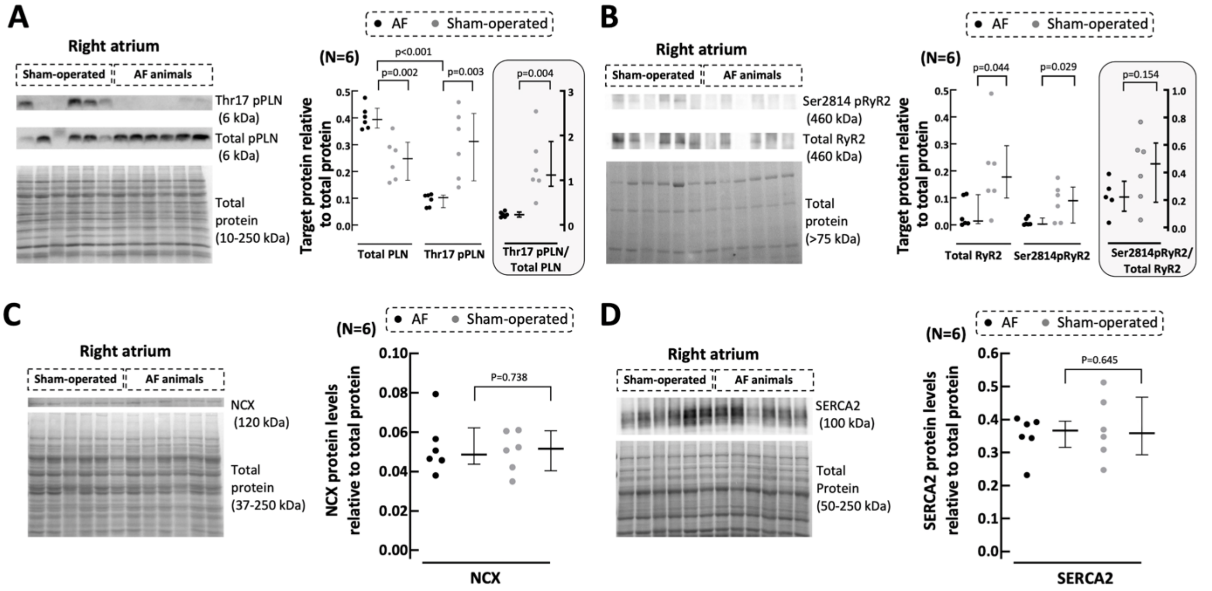

Suppl. Fig. 7. Expression of key calcium handling proteins in the atria of animals with persistent atrial fibrillation and sham-operated controls. Western blot analysis of (A) phospholamban (PLN) and Thr17 phospho-PLN (A), Ryanodine receptor 2 (RyR2) and Ser2814 phospho-RyR2 (B), sodium/calcium exchanger (NCX) (C), and sarco-endoplasmic reticulum ATPase 2 (SERCA2) (D) in right atrial samples from long-lasting persistent atrial fibrillation (AF) animals (N=6) and sham-operated controls (N=6). Bands were quantified relative to total protein and depicted as individual values with median and interquartile range. Two-sided paired/unpaired Student's *t* tests were used to assess differences in phosphorylated/total proteins within the same animals and for group comparisons, respectively. Samples from phosphorylated/total PLN and RyR2 derived from the same experiment and were processed in parallel. Source data are provided as a Source Data file.

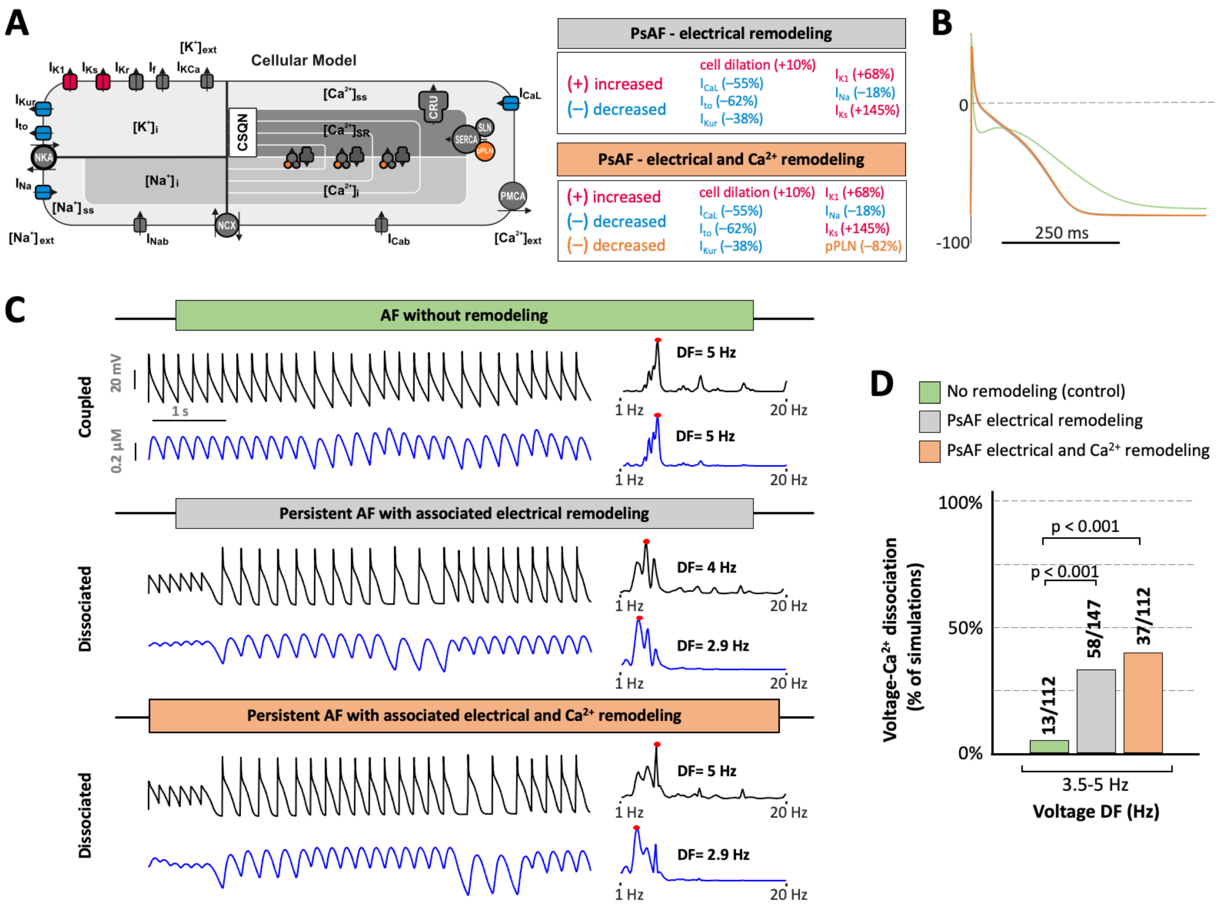

Suppl. Fig. 8. Single cell simulations of human atrial action potentials and its corresponding calcium transients. A, Schematic representation of the cell electrophysiological model (based on Skibsbye *et al*. J Mol Cell Cardiol. 2016;101:26-34). Model parameters highlighted in red, blue and orange represent modifications introduced in the simulation scenarios with atrial fibrillation (AF)-related remodeling. **B**, Simulated action potentials without associated remodeling (in green), persistent AF (PsAF) with associated ion currents remodeling (i.e., electrical remodeling only, in grey) and PsAF with associated electrical and calcium remodeling based on the experimental results (in orange). **C,** Examples of simulated 6-second voltage and calcium signals for the three different scenarios. Their respective power spectral density and dominant frequency (DF) values are also shown. **D,** Prevalence of voltage and calcium dissociation in simulations for each simulated scenario (numbers the indicate the number of simulations runs for each scenario). The Fisher's exact test was used to assess statistical significance. Source data are provided as a Source Data file.

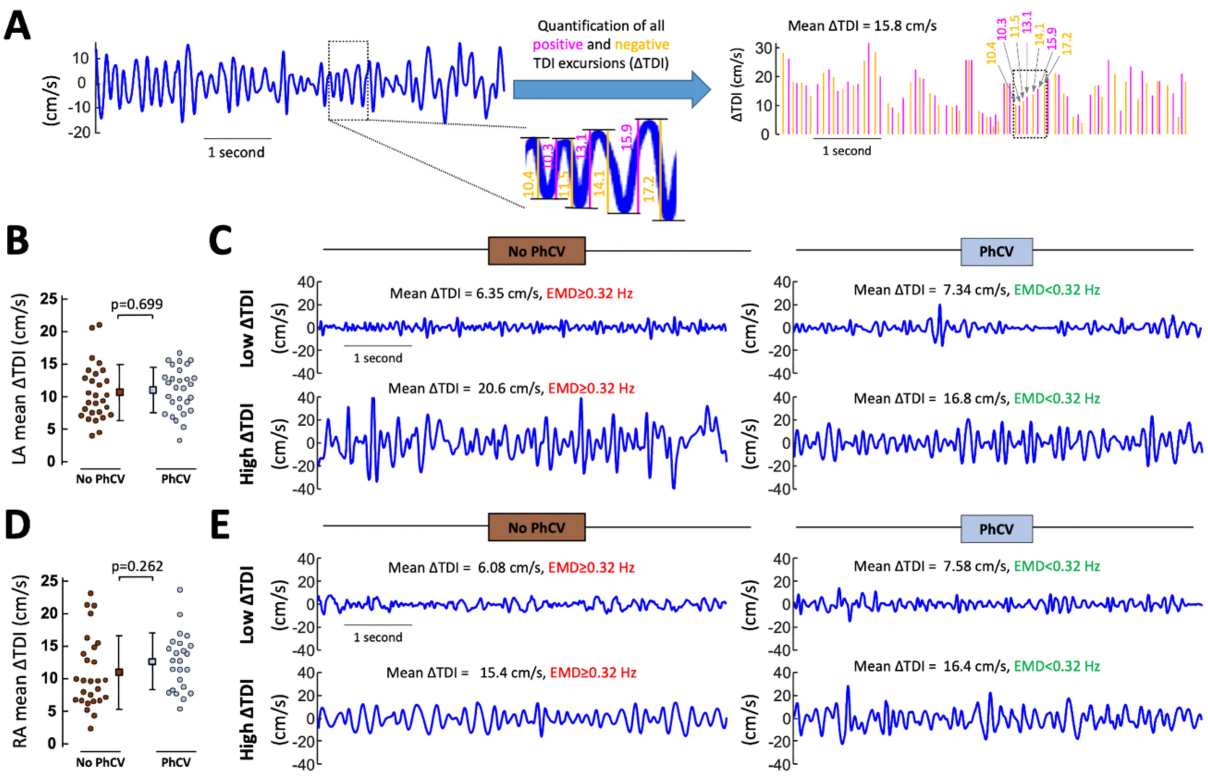

Suppl. Fig. 9. Quantification of amplitude excursion of tissue Doppler imaging signals and prognostic value for the primary outcome. A, Amplitude quantification includes the positive and negative deflections of the tissue Doppler imaging (TDI) signals (in blue). Then, the average value of all amplitude excursions was calculated for each 6-second TDI segment (mean ΔTDI). **B,** Mean ΔTDI in the left atrium (LA) did not differ significantly between patients with successful (n=29) and non-successful (n=28) pharmacological cardioversion (PhCV) within 24 hours after flecainide orally. **C,** Examples of TDI signals from the LA with low/high mean ΔTDI and successful/non-successful PhCV. Unlike electromechanical dissociation values (EMD), the amplitude excursion of TDI signals in the LA was not statistically associated with the primary outcome (PhCV). **D,** Mean ΔTDI in the right atrium (RA) did not differ significantly between patients with successful (n=24) and non-successful (n=27) PhCV. **E,** Examples of TDI signals from the RA with low/high mean ΔTDI and successful/non-successful PhCV. Similar to the LA analysis, the amplitude excursion of TDI signals in the RA was not statistically associated with the primary outcome (PhCV within 24 hours after flecainide orally). All atrial TDI signals (blue tracings) were estimated using intrinsic mode functions (IMFs) 4, 5 and 6 as explained in the Supplemental Methods. In panels B and D, the square and whiskers show mean and standard deviation. Unpaired two-sided *Student's t* tests were used to assess statistical significance. Source data are provided as a Source Data file.

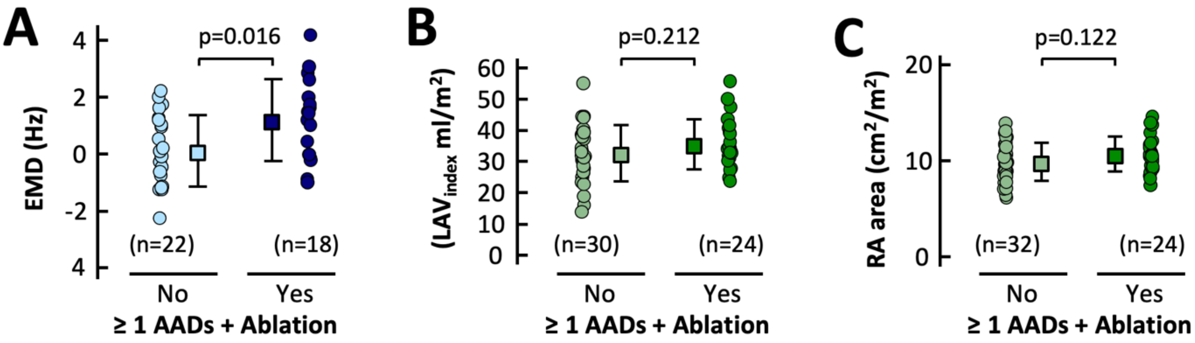

Suppl. Fig. 10. Comparisons of electromechanical dissociation (EMD)(A), Left atrial volume index (LAV_{index}) (B) and right atrial (RA) areas (C) between persistent AF patients treated with ≥1 antiarrhythmic drug (AAD) plus catheter ablation during follow up to attempt rhythm-control and those who did not receive this treatment. The squares and whiskers show mean and standard deviation. Data normality was assessed with the Shapiro-Wilk test. Unpaired two-sided Student's *t* tests were used to assess differences. Source data are provided as a Source Data file.

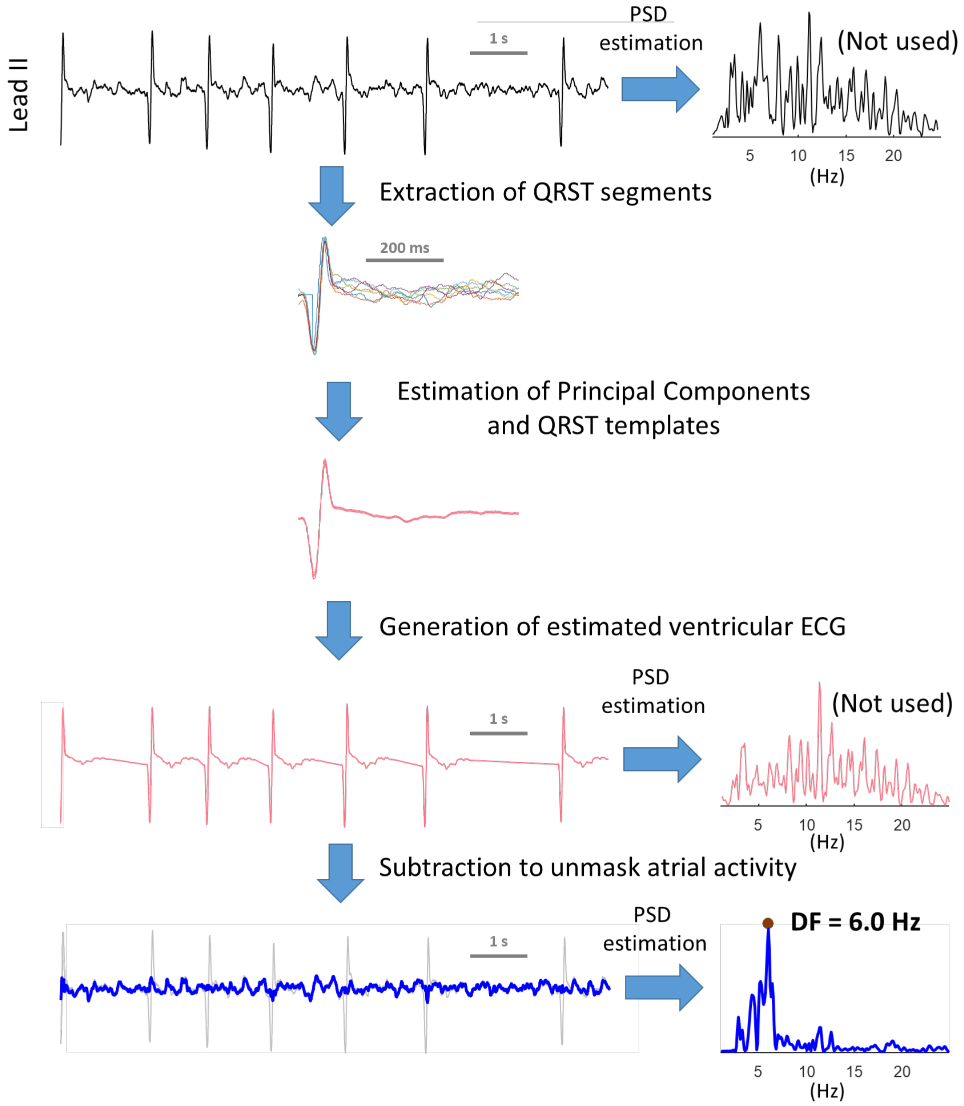

Suppl. Fig. 11. Sample lead II tracing obtained from simultaneous acquisition of tissue Doppler imaging and ECG signals. Principal Component Analysis (PCA) is performed to estimate the ventricular activity (QRS-T complexes) and subtract it from the ECG signal. This process yielded a clean atrial signal on the ECG. The dominant frequency (DF) of the atrial signal was calculated as the frequency with the highest peak in the power spectral density (PSD) within the 3-15 Hz interval. See text for additional details.

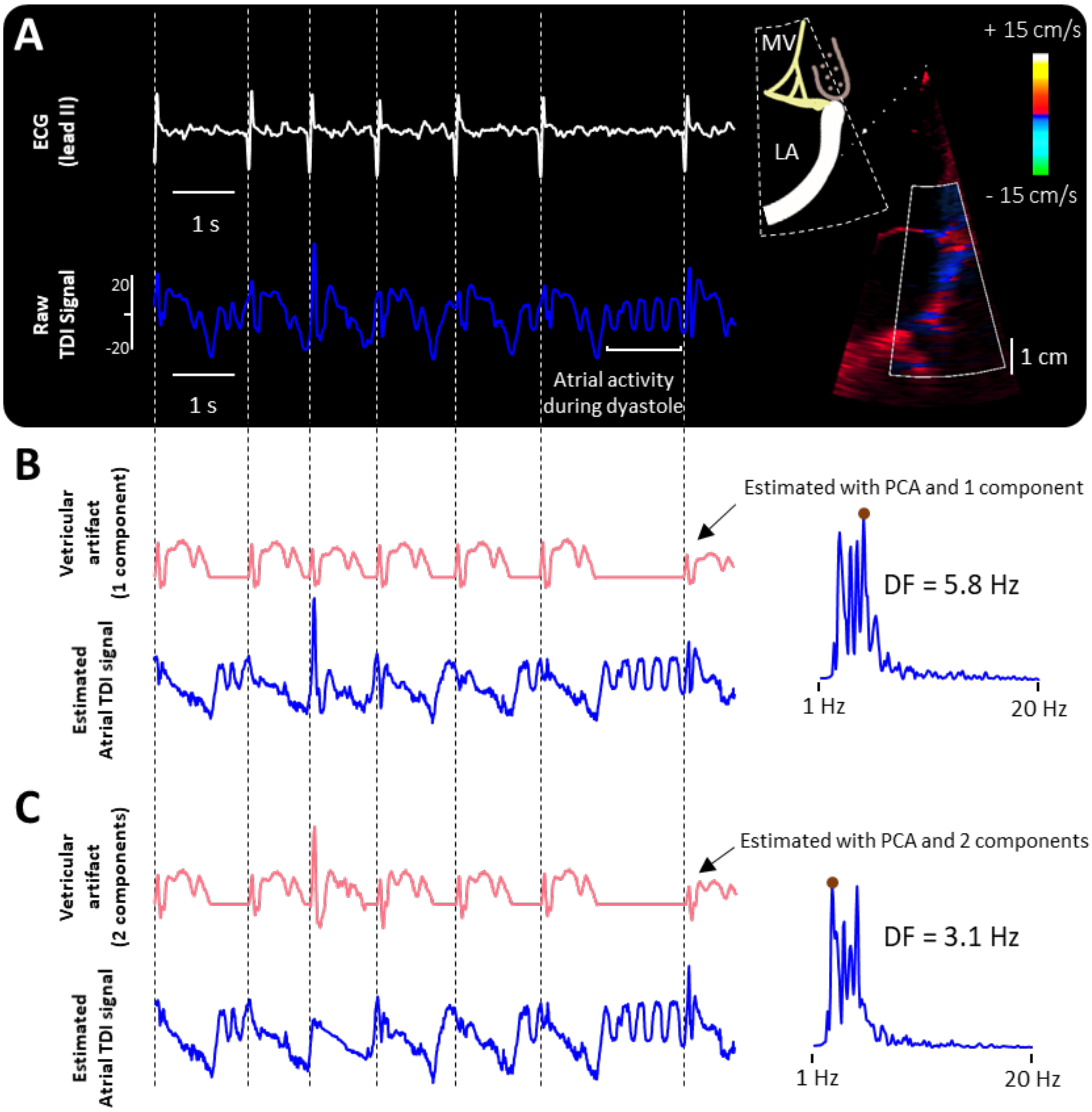

Suppl. Fig. 12. Processing of a tissue Doppler imaging signal from a sample patient using Principal Component Analysis. A, Simultaneous visualization of ECG and TDI signals of the sample human case shown in **Suppl. Fig. 11**. On the right, tissue Doppler imaging (TDI) sequence from left atrium (LA) with the image sector angle on the left atrial wall. A schematic representation of the transthoracic echocardiography view is also shown. **B,** Mechanical ventricular artifacts were estimated with 1 component and then subtracted from the TDI signal using Principal Component Analysis (PCA). The resulting signal was spectrally analyzed to obtain its dominant frequency (DF, on the right). PCA should have yielded a relatively clean atrial mechanical activity. However, this methodology offered a disappointing performance when applied to TDI signals. **C,** The use of 2 components to estimate the mechanical artifact produced by ventricular contraction did not improve the extraction of the underlying mechanical atrial activity on TDI signal. In fact, the latter almost eliminated the underlying atrial activity in the resulting signal. Indeed, the DF of the latter signal was 3.05 Hz (not consistent with the frequency of the atrial mechanical activity in the intervals free from ventricular artifacts). See text for additional details. MV: mitral valve.

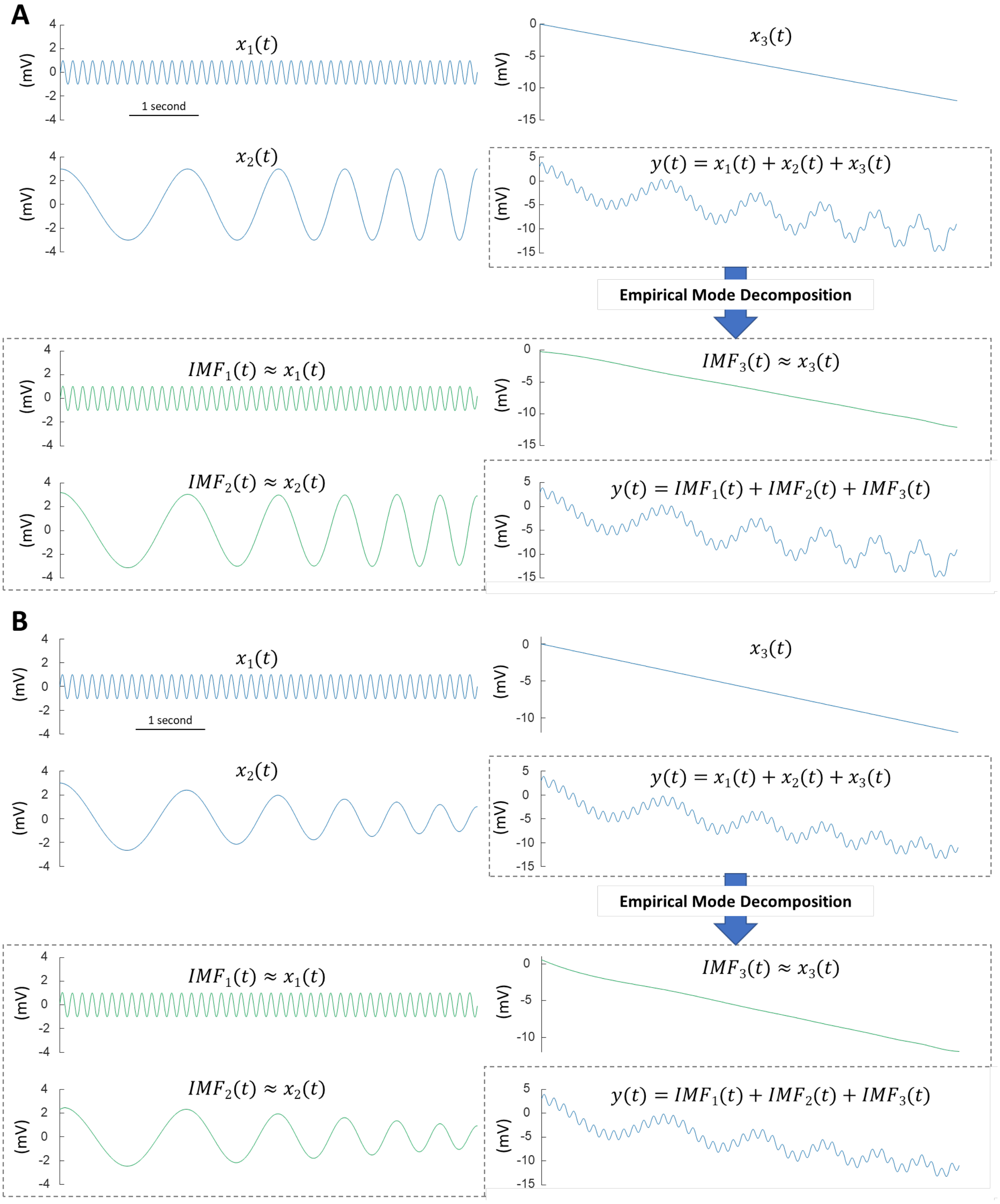

Suppl. Fig. 13. Examples of the performance of the Empirical Mode Decomposition method to separate complex signals into their underlying components (IMFs: intrinsic mode functions). A, synthetic signal obtained as the sum of a 7 Hz sinusoid (roughly simulating a 7 Hz atrial mechanical activity), a sinusoid with a quadratically increasing instantaneous frequency from 0.5 Hz to 2 Hz (roughly simulating a ventricular motion of varying frequency), and a decreasing linear trend. **B,** synthetic signal obtained as the sum of a 7 Hz sinusoid (roughly simulating a 7 Hz atrial mechanical activity), a sinusoid with a quadratically increasing instantaneous frequency from 0.5 Hz to 2 Hz and decreasing amplitude (roughly simulating a ventricular motion of varying frequency), and a decreasing linear trend. Note that in Empirical Mode Decomposition, the sum of all extracted IMFs together yields a perfect reconstruction of the original signal.

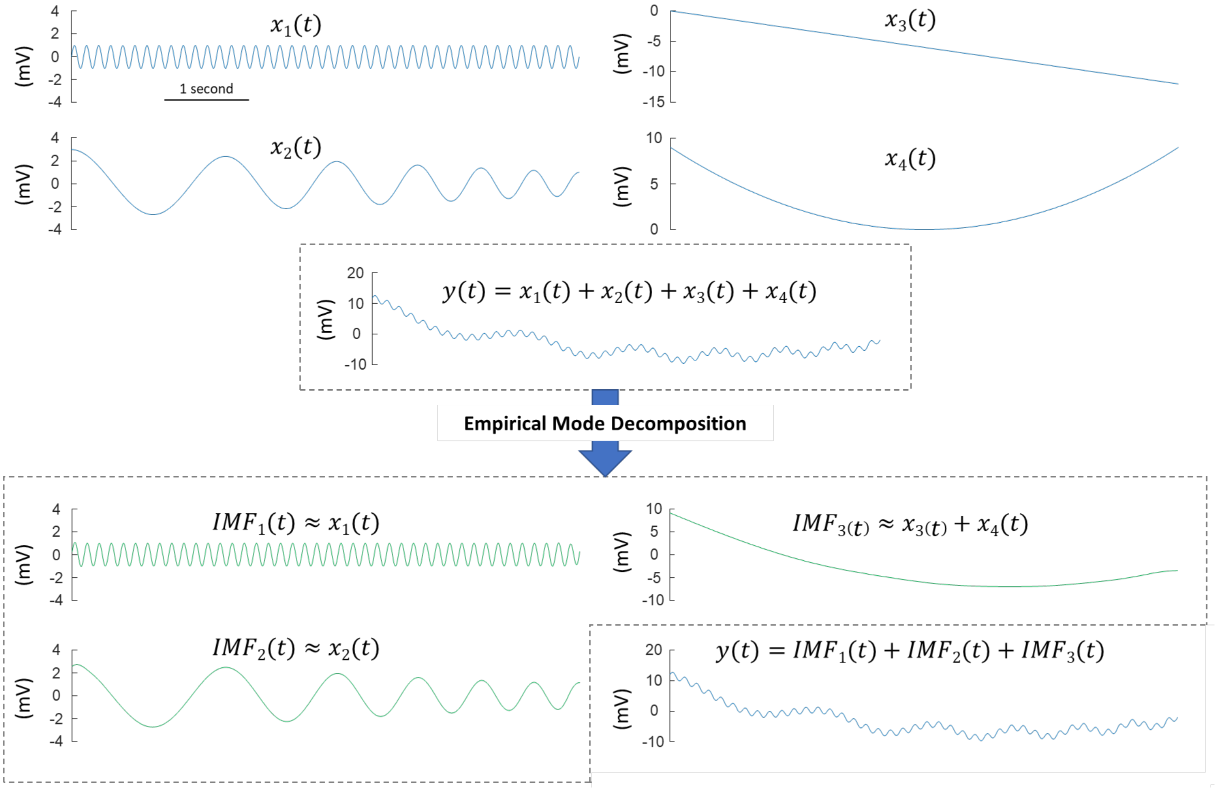

Suppl. Fig. 14. Additional example of the performance of the Empirical Mode Decomposition method to separate a complex signal into their underlying components (IMFs: intrinsic mode functions). A synthetic signal was obtained as the sum of a 7 Hz sinusoid (roughly simulating a 7 Hz atrial mechanical activity), a sinusoid with a quadratically increasing instantaneous frequency from 0.5 Hz to 2 Hz and decreasing amplitude (roughly simulating a ventricular motion of varying frequency), a decreasing linear trend, and a parabolic baseline. In this example, the algorithm successfully extracted the most important components but was unable to separate the linear and parabolic trends. Again, note that the sum of all the IMFs satisfies the perfect reconstruction property.

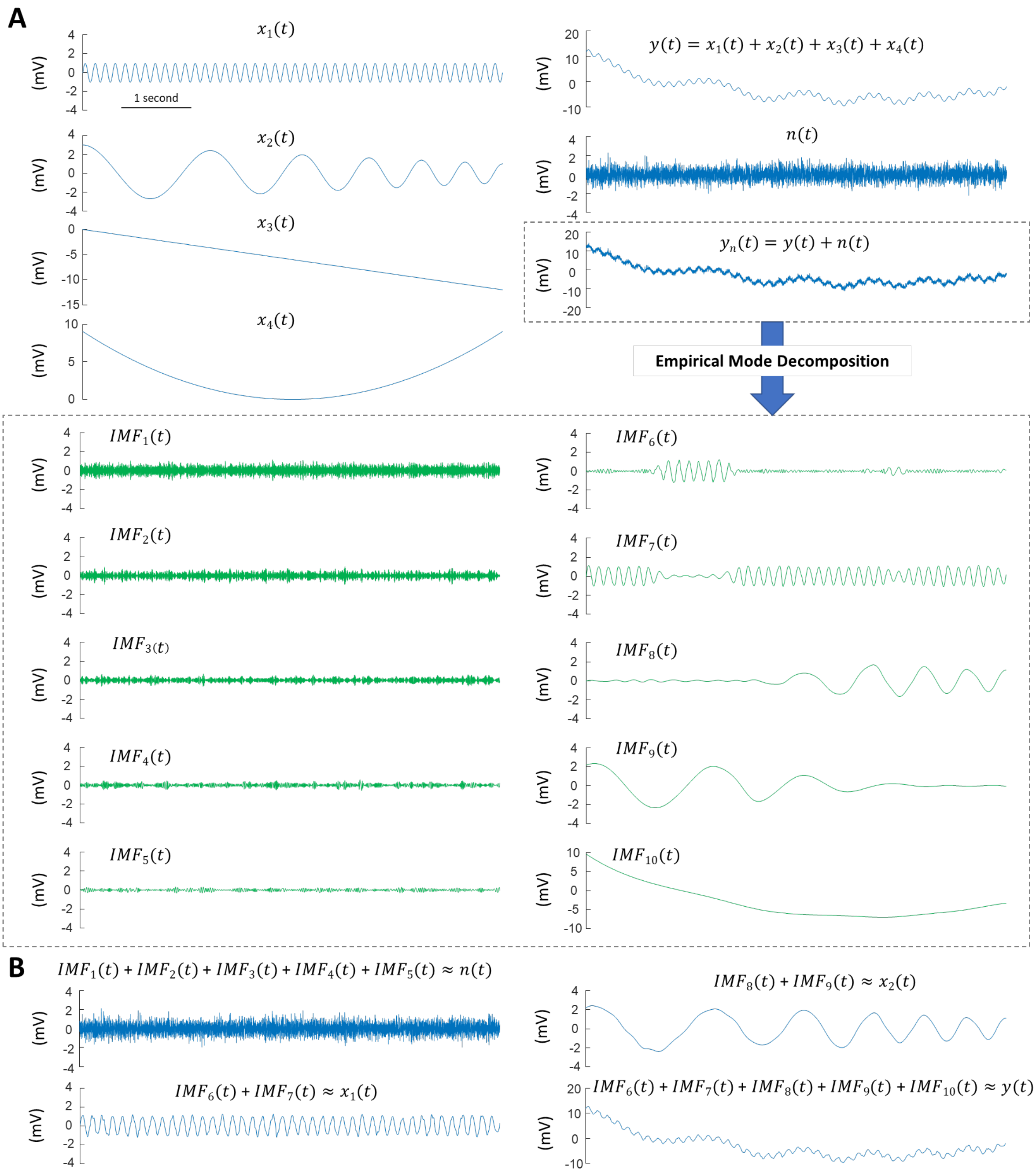

Suppl. Fig. 15. Example of the performance of the Empirical Mode Decomposition method to separate a complex signal into their underlying components (IMFs: intrinsic mode functions) in the presence of noise. A, Additive White Gaussian noise, n(t), with a Signal-to-Noise ratio of 20 dB was added to the signal shown in **Suppl. Fig. 14**. The algorithm decomposed the noisy signal into 10 components. **B,** IMF1-5 were high-frequency noise, IMF6-7 together mostly represented the 7 Hz sinusoid. The combinations of IMFs 8 and 9 approximately yielded the sinusoid with a quadratically increasing instantaneous frequency from 0.5 Hz to 2 Hz and decreasing amplitude. Again, the last IMF was the combination of the linear and quadratic trends. This example highlights the need for adding together several of the IMFs provided by the method to extract meaningful signals in a realistic scenario.

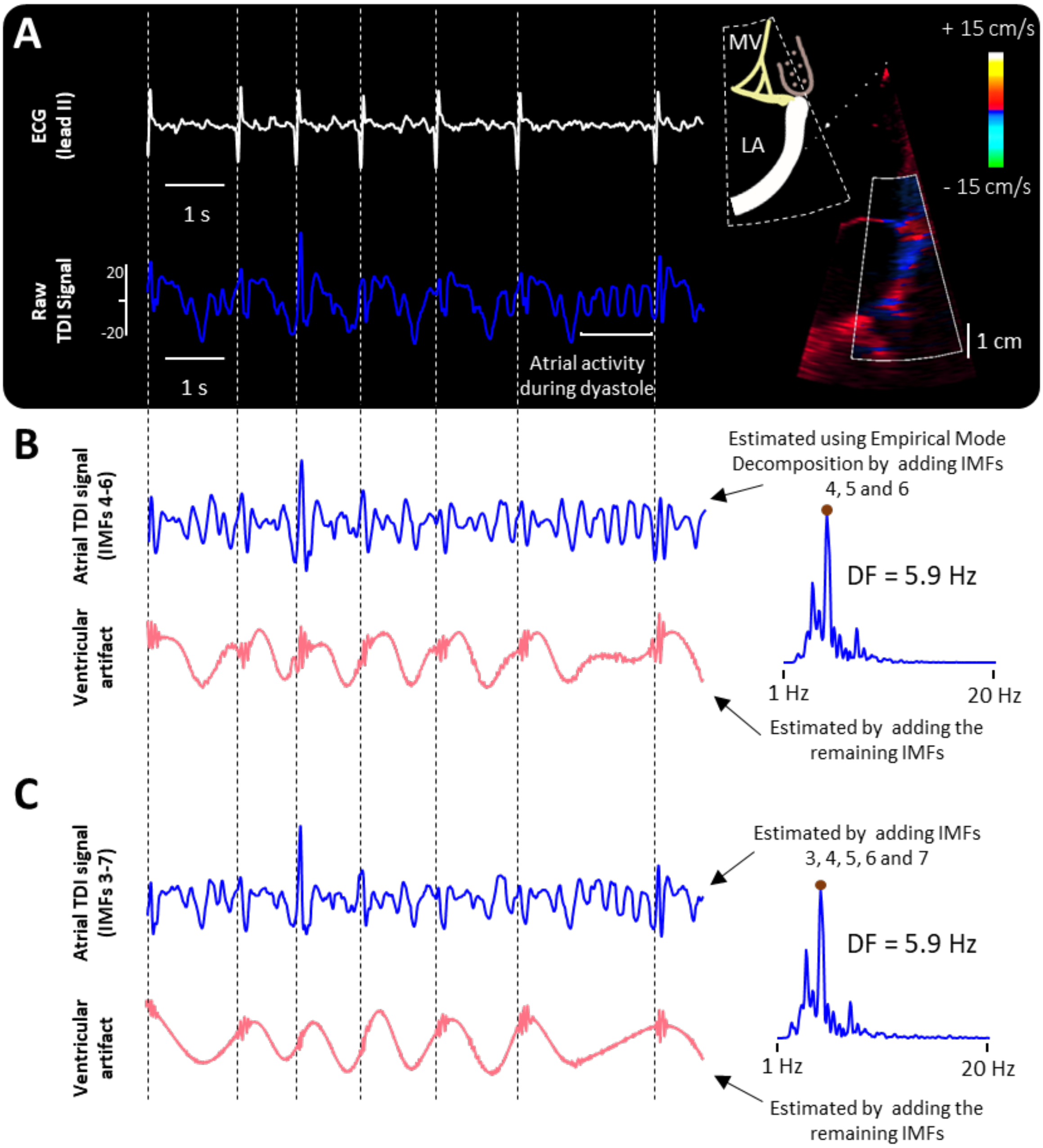

Suppl. Fig. 16. Processing of a tissue Doppler imaging signal from a sample patient using Empirical Mode Decomposition. A, Simultaneous visualization of ECG and TDI signals of the sample human case shown in **Suppl. Fig. 11 and 12**. On the right, TDI sequence from left atrium (LA) with the image sector angle on the left atrial wall. A schematic representation of the transthoracic echocardiography view is also shown. **B,** Atrial TDI signal (blue tracing) estimated by adding intrinsic mode functions (IMFs) 4, 5 and 6. Ventricular motion artifacts (red tracing) were estimated with the remaining IMFs. The Dominant frequency (DF) value of the atrial mechanical signal after spectral analysis is shown on the right. **C,** Reconstruction of the estimated atrial TDI signal (blue tracing) including additional IMFs components below 4 and above 6 did not change substantially neither the shape of the spectrum nor the obtained DF value. Interestingly, Empirical Mode Decomposition offered a considerably better performance than Principal Component Analysis (PCA) when applied to TDI signals. See text for additional details. MV: mitral valve.

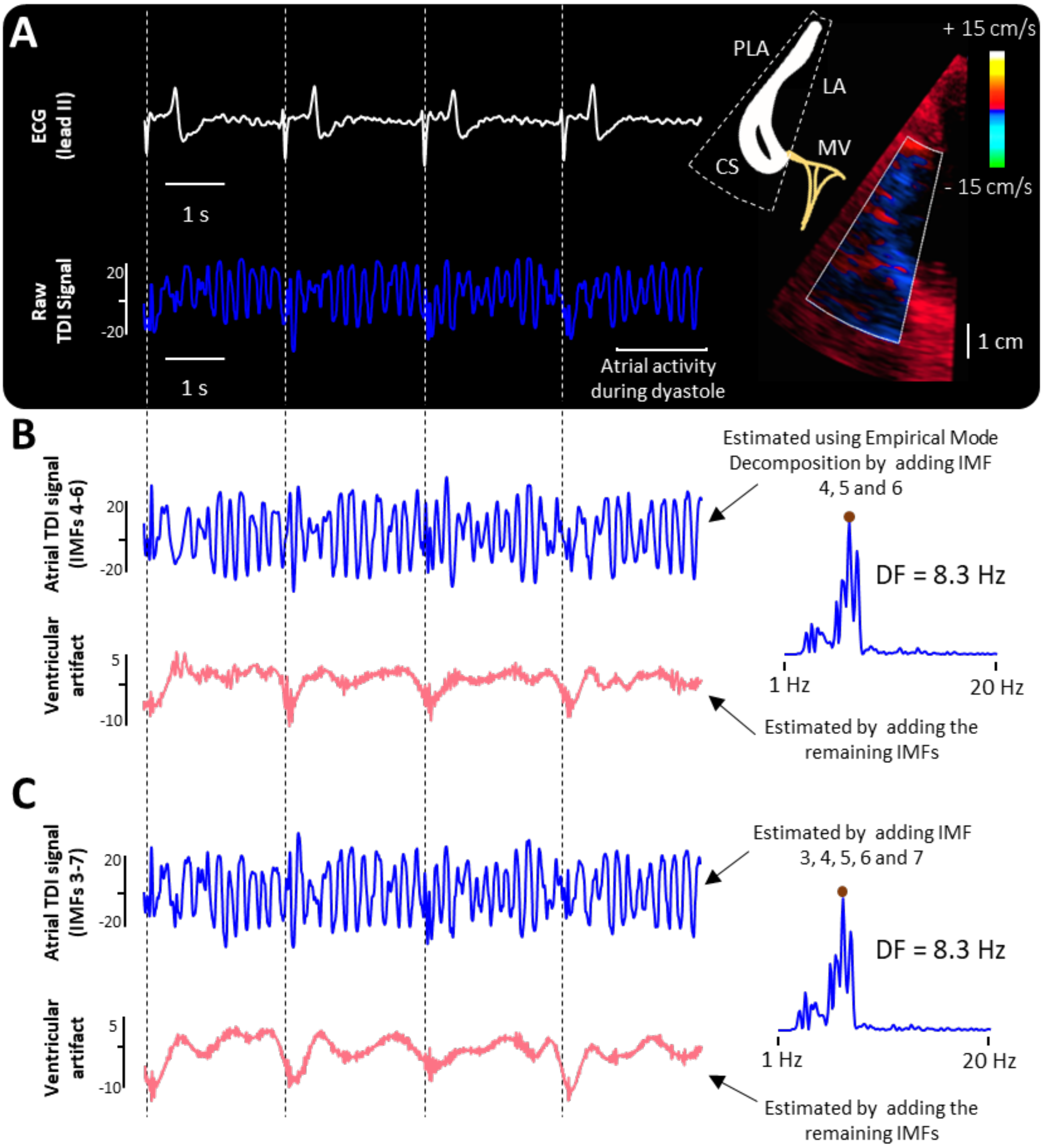

Suppl. Fig. 17. Processing of a tissue Doppler imaging signal from a sample pig using Empirical Mode Decomposition. A, Simultaneous visualization of ECG and **tissue Doppler imaging** (TDI) signals using transesophageal echocardiography. On the right, TDI sequence from left atrium (LA) with the image sector angle on the posterior left atrial wall (PLA). A schematic representation of the transesophageal echocardiography view is also shown. **B,** Atrial TDI signal (blue tracing) estimated by adding intrinsic mode functions (IMFs) 4, 5 and 6. The residual artifacts (red tracing) were estimated by adding the remaining IMFs. The latter was mainly associated with ventricular motion artifacts but also with high frequency components similar to the simulated white gaussian noise in **Suppl. Fig. 15.** The Dominant frequency value of the atrial mechanical signal after spectral analysis is shown on the right. **C:** Reconstruction of the estimated atrial TDI signal including additional IMFs components below 4 and above 6 did not change substantially neither the shape of the spectrum nor the obtained DF value. CS: coronary sinus. MV: mitral valve.

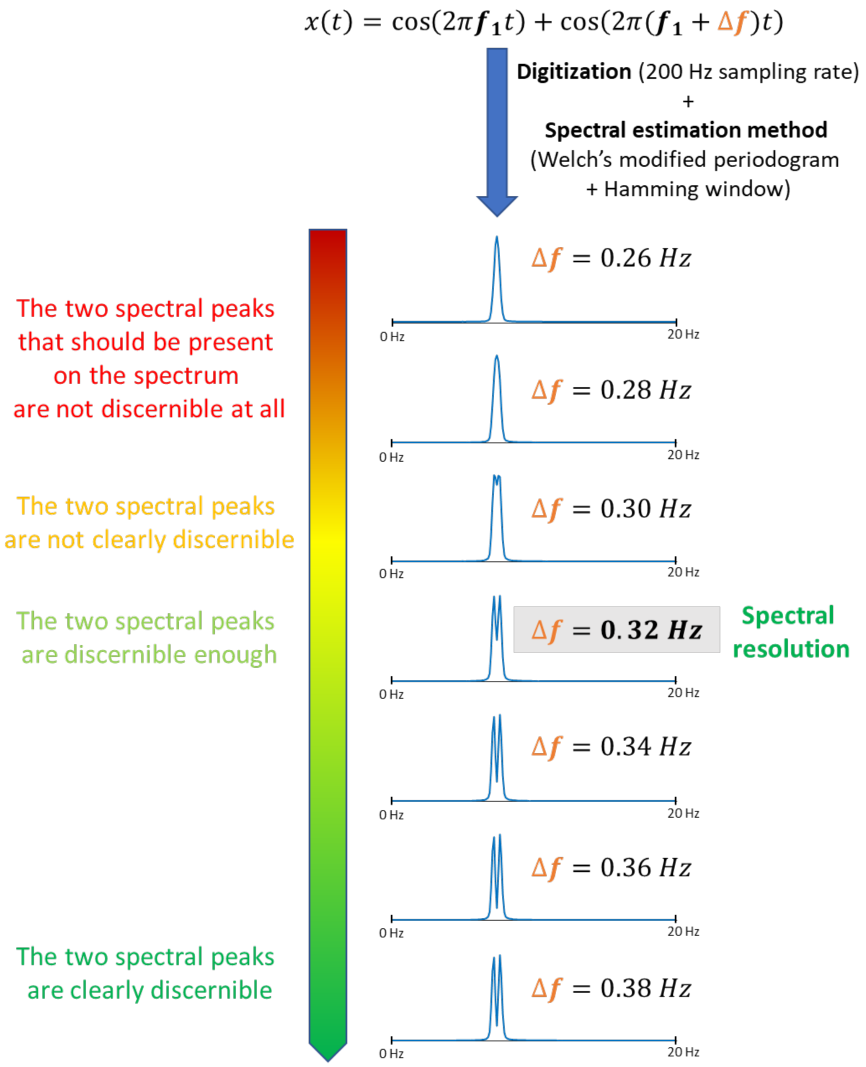

Suppl. Fig. 18. Determination of the spectral resolution of the power spectral density estimation method used to assess electromechanical dissociation during atrial fibrillation *in vivo*. A Welch's periodogram of the signals previously multiplied by a Hamming window and zero-padded to the next higher power of 2 was used as power spectral density (PSD) estimation method for both ECG and tissue Doppler imaging (TDI) signals. Signals were acquired simultaneously with a sampling rate of at least 200 Hz. Using our methodology, two sinusoidal signals, cos($2\pi \boldsymbol{f_1}$) and cos($2\pi(\boldsymbol{f_1}+\Delta \boldsymbol{f})$), with slightly different frequencies ($\boldsymbol{f_1}$ and $\boldsymbol{f_1}+\Delta \boldsymbol{f}$) would not be discernable in the frequency spectrum and would be displayed as a single fused spectral lobe unless $\Delta \boldsymbol{f}$ >0.32 Hz. Therefore, the minimal $\Delta \boldsymbol{f}$ above which two distinct spectral lobes start to be discernible enough (i.e., spectral resolution) was 0.32 Hz with the PSD estimation method used in this study for signal processing. That was the value we used as the minimal difference between the dominant frequencies (DF) of ECG and TDI signals for considering that electromechanical dissociation (EMD) was actually present.

Suppl. Table 1. Proteins with statistically significant changes over the sequential biopsy studies.

Gene Ontology	Included genes
**Muscle Contraction**	ACTA1 / ACTC1 / CAV3 / TNNT2 / TPM1 / TNNI1 / ALDOA / CRYAB
**Collagen-containing extracelular matrix**	FBN2 / SDC2 / ANXA4 / ADIPOQ / LTBP4 / BGN / FBLN1 / AEBP1 / HSP90B1 / HNRNPM / VCAN / ABI3BP / COL5A2 / MFAP2 / SERPINH1 / COL6A6 / FBN1
**Extracellular matrix structural constituent conferring compression resistance**	LUM / DCN / OGN / BGN / PRELP / ASPN
**Others**	ABCB10 / ABCD3 / ABCF1 / ABHD12 / ACACB / ACADL / ACADM / ACADVL / ACBD5 / ACSS2 / ACTR1B / ADK / ADSL / ADSSL1 / AHCY / AIDA / AK1 / AK2 / AKAP6 / AKAP8 / ALDH1A2 / ALDH4A1 / AMACR / ANO10 / ANXA11 / ANXA7 / AP1B1 / AP1M1 / AP3B1 / APOBEC2 / appl2 / ARCN1 / ARF3 / ARF4 / ARGLU1 / ARHGAP35 / ARMCX3 / ART3 / ASNS / ASPH / ATOX1 / ATP13A1 / ATP1A1 / ATP1B1 / ATP4A / ATPAF2 / AUP1 / BAG2 / BDH1 / BIN1 / BSG / BZW1 / C12orf57 / C16orf58 / C8orf82 / CABP1 / CAND1 / CAPZA2 / CARD19 / CBX3 / CCDC124 / CCDC141 / CCDC47 / CCDC58 / CCPG1 / CCT2 / CCT6B / CCT7 / CD81 / CDH13 / CHCHD6 / CHORDC1 / CHTOP / CLASP2 / CLTC / CMYA5 / COA3 / COPG1 / COPS2 / COQ10A / CORO2B / CPNE3 / CRELD1 / CRELD2 / CRYZ / CSDE1 / CSNK2A1 / CSTF2 / CTNNA1 / CTPS2 / CTSD / CUX1 / CYC1 / DAZAP1 / DBH / DCPS / DDX10 / DECR1 / DES / DHRS7C / DIABLO / DIAPH2 / DMAP1 / DMD / DNAJA4 / DNAJB6 / DNAJC19 / DOCK1 / DPP3 / DR1 / DTYMK / DYSF / ECHDC2 / EEF1E1 / EHD3 / EHD4 / EIF3H / EIF4A2 / EIPR1 / EMC2 / EMC4 / ENAH / ENAH / ENPP1 / EPB41L3 / EPHX1 / ERCC3 / ETFDH / FAM111A / FAM114A1 / FAM162A / FAM185A / FAM98A / FBXO3 / FBXO30 / FCN2 / FITM2 / FKBP5 / FLNC / FNDC3B / GALK1 / GALNT2 / GAPDH / GAPDHS / GFAP / GHITM / GPD1 / GPD1L / GPI / GTPBP1 / GTPBP3 / GYG1 / H2AFZ / HADHA / HADHB / HAGH / HAX1 / HEBP1 / HEXA / HHATL / HIGD1A / Higd2a / HIST1H1B / HIST1H1D / HK1 / HK2 / HMGN1 / HMOX2 / HNRNPA1 / HNRNPDL / HNRNPH1 / HNRNPH1 / HNRNPUL2 / HPRT1 / HSD17B14 / HSDL2 / HSPA12B / HYI / IDH2 / IDH3B / IFIT3 / IFIT5 / IGHG / IGHG / IGHG / IKBIP / ILF3 / INPP1 / ITGA5 / ITGB5 / ITIH3 / KBTBD3 / KDSR / KLC2 / KPNA1 / KTN1 / KYAT1 / LAMA2 / LAMB2 / LAMTOR4 / LAP3 / LDB3 / LDHB / LDHB / LETM1 / LGMN / lias / LMNA / LMNB1 / LMNB2 / LMO7 / LOC100157017 / LOC100158003 / LOC100512341 / LOC100622780 / LOC100736765 / LOC102164667 / LOC102165015 / LOC110257570 / LPCAT3 / LRBA / LTF / LYNX1 / LZIC / MAN2B1 / MANF / MAOA / MAP2K1 / MAP4 / MAPK1IP1L / MASP1 / MATR3 / MDP1 / MECP2 / MIA2 / MLEC / MLF1 / MOB2 / MRPL1 / MRPL19 / MRPL2 / MRPS18A / MSRB2 / MSRB3 / MT-ND5 / MTX1 / MVP / MX2 / MXRA7 / MYBBP1A / MYL1 / MYL3 / MYO18A / MYO1C / MYO1D / MYOZ2 / NCEH1 / NDRG2 / NDRG4 / NDUFA4L2 / NDUFAF3 / NES / NFIA / NGLY1 / NMNAT3 / NMT1 / NOP58 / NPPB / NT5C2 / NUCB1 / NUCB2 / NUMA1 / NUP62 / OLFML1 / OSBP / OTULIN / PAFAH1B3 / PALMD / PARP1 / PBLD / PDIA5 / PDK2 / PDK4 / PDLIM1 / PDLIM5 / PDXDC1 / PECR / PFKM / PGK1 / PGM2 / PGPEP1 / PICALM / PITPNB / PITPNB / PLVAP / PMPCB / PON2 / PPM1E / PPME1 / PPP1R12C / PPP6R2 / PRDX6 / PRELP / PRKAG1 / PRPF40A / PRPF6 / PRPH / PRUNE1 / PRXL2A / PSMB3 / PSMC2 / PSMC3 / PSMC4 / PSMC5 / PSMD1 / PSMD13 / PSMD3 / PSME2 / PSPC1 / PTPN9 / PUM1 / PURB / PYGB / PYGB / PYGM / QKI / RAB12 / RAB7A / RAD21 / RASIP1 / RBBP4 / RBM24 / RBM25 / RBM39 / RBM4B / RBMX / RECK / RICTOR / RNF146 / RPGR / RPN1 / RPN2 / RPS10 / RPS27 / RPS6KA5 / RTCA / RTRAF / S100A1 / SACS / SAE1 / SAFB / SCARB2 / SCCPDH / SCP2 / SCYL2 / SDR39U1 / SEPT11 / SERBP1 / SERHL2 / SFPQ / SH3BGRL / SH3GL2 / SLA-2 / SLC25A20 / SLC25A46 / SLC25A6 / SLC9A1 / SLK / SLMAP / SMPDL3B / SMYD1 / SNAP23 / SNF8 / SNRPC / SNRPGP15 / SNX5 / SORBS2 / SORBS2 / SPAG9 / SPATS2L / SPON1 / SPR / SPRYD7 / SRR / SRSF4 / SRSF5 / SRSF7 / SSBP1 / SSRP1 / STAMBP / STOML2 / STT3A / STUB1 / STX7 / STXBP1 / STXBP3 / SUGCT / SUGP2 / SUOX / SURF1 / SYNPO2L / TBC1D1 / TBCD / TBX20 / TCP1 / TGFBI / TGM2 / THAP8 / THNSL2 / THRAP3 / THSD4 / THSD7A / TIMM10B / TIMM17B / TM9SF4 / TMED10 / TMED9 / TMEM143 / TMEM33 / TMEM70 / TMEM97 / TNS2 / TNXB / TOMM5 / TOP2B / TP53BP1 / TRAP1 / TRIM25 / TSTD1 / TSTD3 / TTC32 / TUBA4A / TXNDC5 / TXNIP / TXNRD1 / UACA / UBE2D3 / UBE2K / UBTF / UQCC / UQCRC1 / UROD / USO1 / USP7 / VAC14 / VAPB / VAT1L / VDAC3 / VLDLR / VPS35L / VSNL1 / WASHC1 / WDR1 / WWC3 / XPNPEP2 / YME1L1 / YWHAH / YWHAZ / ZC2HC1A / ZNF148 / ZNF44 / ZSWIM8

Suppl. Table 2. Gene primers to amplify cDNA in RT-qPCR reactions

Gene	Forward primer (5'-3')	Reverse primer (5'-3')
lox	cacagcatacagggcagatg	ccatgctgtggtaatgttgg
gapdh	ccatcttccaggagcgagat	agaaggggcagagatgatga

Suppl. Table 3. Concentrations and references for the all the antibodies used in the study.

Antibody	Concentration	Source	Reference
ACTC1	1:10000	Mouse	66125-1-Ig (ProteinTech®)
BAX	1:1000	Rabbit	M00183-1 (Boster Bio)
MYBPC3		Mouse	Sc-137237 (Santa Cruz Biotechnology)
NCX	1:1000	Mouse	MA3-926 (Thermo Fisher)
PLN	1:5000	Mouse	A010-14 (Badrilla)
pThr17 PLN	1:5000	Rabbit	A010-13 (Badrilla)
RyR2	1:1000	Mouse	MA3-916 (Thermo Fisher)
pSer2814 RyR2	1:2000	Rabbit	A010-31 (Badrilla)
SERCA2	1:1000	Mouse	Sc-376235 (Santa Cruz Biotechnology)
Polyclonal Goat Anti-Mouse (Ig/HRP)	1:10000	Goat	P044701-2 (Dako)
Goat anti-rabbit (Ig/HRP)	1:10000	Goat	P044801-2 (Dako)
