# Supplementary material for: Non-invasive electromechanical assessment during atrial fibrillation identifies underlying atrial myopathy alterations with early prognostic value
Source: Nat Commun. 2023 Aug 4;14:4613. doi: 10.1038/s41467-023-40196-y (PMC10403561; doi:10.1038/s41467-023-40196-y)
Supplement: Supplementary file 3 — Description of Additional Supplementary Files [file 41467_2023_40196_MOESM3_ESM.pdf]

**Description of Additional Supplementary Files**

**Supplementary Movie 1. Sample transthoracic echocardiography study in a 52-year-old patient with persistent atrial fibrillation.** The left panel shows a tissue Doppler imaging (TDI) movie focused on the left atrial wall. The movie shows simultaneous acquisition of led II ECG tracings (bottom). The right panel shows left atrial wall tracking based on echogenicity criteria. Color-coded wall tracking shows 3 echogenicity thresholds (red: 75%, green: 60% and blue: 30% of the maximum echogenicity). The echogenicity threshold for wall tracking and subsequent TDI signal retrieval for analysis was set at 30% of the maximum echogenicity within the atrial region of interest. Additional data from this patient is shown in Fig. 8A.

**Supplementary Movie 2. Sample transesophageal echocardiography study in the 52-year-old patient with persistent atrial fibrillation shown in Suppl. Movie 1.** The left panel shows a tissue Doppler imaging (TDI) movie focused on the left atrial wall. The movie shows simultaneous acquisition of led II ECG tracings (bottom). The right panel shows left atrial wall tracking based on echogenicity criteria. Color-coded wall tracking shows 3 echogenicity thresholds (red: 75%, green: 60% and blue: 30% of the maximum echogenicity). The echogenicity threshold for wall tracking and subsequent TDI signal retrieval for analysis was set at 30% of the maximum echogenicity within the atrial region of interest. Additional data from this patient is shown in Fig.8A.

**Supplementary Movie 3. Sample transesophageal echocardiography study during the follow-up of a pig with atrial fibrillation.** The left panel shows a tissue Doppler imaging (TDI) movie focused on the posterior left atrial wall. The movie shows simultaneous acquisition of led II ECG tracings (bottom). The right panel shows posterior left atrial wall tracking based on echogenicity criteria. Color-coded wall tracking shows 3 echogenicity thresholds (red: 75%, green: 60% and blue: 30% of the maximum echogenicity). The echogenicity threshold for wall tracking and subsequent TDI signal retrieval for analysis was set at 30% of the maximum echogenicity within the atrial region of interest.
