# Supplementary material for: Non-invasive electromechanical assessment during atrial fibrillation identifies underlying atrial myopathy alterations with early prognostic value
Source: Nat Commun. 2023 Aug 4;14:4613. doi: 10.1038/s41467-023-40196-y (PMC10403561; doi:10.1038/s41467-023-40196-y)
Supplement: Supplementary file 7 — Reporting Summary [file 41467_2023_40196_MOESM7_ESM.pdf]

nature portfolio

Corresponding author(s): Filgueiras-Rama David

Last updated by author(s): 2023/07/15

Reporting Summary

Nature Portfolio wishes to improve the reproducibility of the work that we publish. This form provides structure for consistency and transparency in reporting. For further information on Nature Portfolio policies, see our Editorial Policies and the Editorial Policy Checklist.

Statistics

For all statistical analyses, confirm that the following items are present in the figure legend, table legend, main text, or Methods section.

n/a	Confirmed	
☐	☒	The exact sample size (*n*) for each experimental group/condition, given as a discrete number and unit of measurement
☐	☒	A statement on whether measurements were taken from distinct samples or whether the same sample was measured repeatedly
☐	☒	The statistical test(s) used AND whether they are one- or two-sided<br>*Only common tests should be described solely by name; describe more complex techniques in the Methods section.*
☐	☒	A description of all covariates tested
☐	☒	A description of any assumptions or corrections, such as tests of normality and adjustment for multiple comparisons
☐	☒	A full description of the statistical parameters including central tendency (e.g. means) or other basic estimates (e.g. regression coefficient) AND variation (e.g. standard deviation) or associated estimates of uncertainty (e.g. confidence intervals)
☐	☒	For null hypothesis testing, the test statistic (e.g. *F*, *t*, *r*) with confidence intervals, effect sizes, degrees of freedom and *P* value noted<br>*Give P values as exact values whenever suitable.*
☒	☐	For Bayesian analysis, information on the choice of priors and Markov chain Monte Carlo settings
☒	☐	For hierarchical and complex designs, identification of the appropriate level for tests and full reporting of outcomes
☐	☒	Estimates of effect sizes (e.g. Cohen's *d*, Pearson's *r*), indicating how they were calculated

Our web collection on statistics for biologists contains articles on many of the points above.

Software and code

Policy information about availability of computer code

Data collection	All custom-written code, graphical user interfaces and examples of test data are available at https://gitlab.com/Advanced_Development_in_Arrhythmia_Mechanisms_and_Therapy_Lab/Electromechanical_Assessment_During_AF. Other comercial software used for data collection was: Matlab (R2016b), EnSite Precision Cardiac Mapping System, iE33 ultrasound system (software-6.0.0.845), iE33 ultrasound system (software-6.3.7.745), Epiq7 system (software-1.8.6). All commercial software (including the version) is specifically mentioned in the main manuscript and Suppl. Material.
Data analysis	All custom-written code, graphical user interfaces and examples of test data are available at https://gitlab.com/Advanced_Development_in_Arrhythmia_Mechanisms_and_Therapy_Lab/Electromechanical_Assessment_During_AF. Other comercial software used for data analysis was: Matlab (R2016b),Open access Fiji ImageJ, Stata/IC 15.1 and RStudio (3.5.2). All commercial software (including the version) is specifically mentioned in the main manuscript and Suppl. Material.

For manuscripts utilizing custom algorithms or software that are central to the research but not yet described in published literature, software must be made available to editors and reviewers. We strongly encourage code deposition in a community repository (e.g. GitHub). See the Nature Portfolio guidelines for submitting code & software for further information.

Data

Policy information about availability of data

All manuscripts must include a data availability statement. This statement should provide the following information, where applicable:

- Accession codes, unique identifiers, or web links for publicly available datasets
- A description of any restrictions on data availability
- For clinical datasets or third party data, please ensure that the statement adheres to our policy

Source data are provided with this paper. All custom-written code, graphical user interfaces, Stata scripts for logisitic models and examples of test data are available at https://gitlab.com/Advanced_Development_in_Arrhythmia_Mechanisms_and_Therapy_Lab/Electromechanical_Assessment_During_AF. All data needed to reproduce the study can be found in the manuscript, supplementary information and source data. The mass spectrometry proteomics data are available via ProteomeXchange with identifier PXD043016.

Research involving human participants, their data, or biological material

Policy information about studies with human participants or human data. See also policy information about sex, gender (identity/presentation), and sexual orientation and race, ethnicity and racism.

Reporting on sex and gender	Sex was considered in the study design. Sex was included as a clinical variable. Sex did not any statistically significant association with the primary outcome. The results may apply to both sexes, although larger series may be necessary to specifically address sex differences on electromechanical remodeling. Sex was assigned based on medical records. Gender was not specifically collected. Individual patient data cannot be shared without permission of the study participants and ethical approval.
Reporting on race, ethnicity, or other socially relevant groupings	We did not specifically study the associations of race, ethnicity or other socially groups on study outcomes. The study sample size is also limited to address any effect related to race, ethnicity or other social groups.
Population characteristics	The clinical cohort consisted of a prospective series of patients admitted to 2 tertiary hospitals from October 2014 to March 2022 with a recent atrial fibrillation (AF) diagnosis. The study was designed to include patients <70 years old, at early remodeling stages of AF progression (paroxysmal AF or persistent with ≤6 months of episode duration based on symptoms) and lack of other relevant comorbidities, significant structural heart disease or baseline treatment with antiarrhythmic drugs, other than ß-blockers. More specifically, patients taking amiodarone within the 6-month period before inclusion, or other antiarrhythmic drugs within the month before inclusion, were excluded. All patients were free of heart failure symptoms and other signs of severe cardiac or systemic comorbidities on physical examination, ECG, X-ray, and routine blood testing. After admission, patients with left ventricular ejection fraction <50% or any documented significant valvulopathy (moderate–severe stenosis or regurgitation) were excluded from the study at the time of conventional transthoracic echocardiography. We also excluded patients with left atrial volumes >55 ml/m2 due to the low probability of effective rhythm control during the follow-up The study population (N=83) was composed of relatively young patients (55.8±9.8 years old) with low rates of comorbidities (See Table 1 for details) and average 3D left atrial volume index values (32.6±8.6 ml/m2) below the dilation threshold (34 ml/m2). In 65% of patients (n=54), the AF episode was the first one detected, and only 12 patients had an AF history with ≥2 episodes. The majority (71%) of patients with persistent AF had no previous AF history. Episode duration before attempting pharmacological cardioversion was 18 hours [interquartile range: 10.0, 30.0 hours] in paroxysmal AF patients and 60.0 days [interquartile range: 16.0, 121.7 days] in patients with persistent AF. Altogether, these data indicate an AF population at early remodeling stages, which minimized the risk of potential confounding factors affecting atrial remodeling beyond AF itself.
Recruitment	The clinical cohort consisted of a prospective series of patients admitted to 2 tertiary hospitals from October 2014 to March 2022 with a recent atrial fibrillation (AF) diagnosis. A total of 66 consecutive patients with persistent AF and 31 more consecutive patients with paroxysmal AF were invited to participate (77% from the emergency department, 23% from the arrhythmia office). Patient recruitment and inclusion criteria were designed to include patients at early stages of AF progression. Recruitment with more general criteria would have included patients with advanced stages of atrial myopathy. Therefore, the study population should be considered a population with low probability of confounding factors aiming to understand AF-related remodeling, which may not apply for patients with atrial myopathy secondary to other diseases.
Ethics oversight	The study in patients was approved by the ethics committees of the Hospital Clínico Universitario San Carlos (Ref#14/273-E) and the Hospital Universitario Central de Asturias (Ref#07/16). All patients gave written informed consent.

Note that full information on the approval of the study protocol must also be provided in the manuscript.

Field-specific reporting

Please select the one below that is the best fit for your research. If you are not sure, read the appropriate sections before making your selection.

☒ Life sciences ☐ Behavioural & social sciences ☐ Ecological, evolutionary & environmental sciences

For a reference copy of the document with all sections, see nature.com/documents/nr-reporting-summary-flat.pdf

Life sciences study design

All studies must disclose on these points even when the disclosure is negative.

Sample size	We initially calculated a minimum sample size of 50 patients to detect ≥0.7 Hz differences between mechanical and electrical activations rates. This was done assuming an alpha risk 0.05 and Beta risk 0.2. Differences were based on available data on electrical remodeling progresssion from paroxysmal to persistent atrial fibrillation in animal models. However, no data were available about the relationship between mechanical and electrical activation rates for more accurate estimations of the sample size at the beggining of the clinical study in 2014. Therefore, our initial sample sample size estimations should be considered as those for pilot studies.
Data exclusions	All available data were included in the analyses. No data were excluded from the analysis.
Replication	The study protocol is described in the main manuscript. Two participant centers reproduced the same protocol. Imaging and study data were prospectively collected in a consistent manner among participants. Investigators involved in imaging and data analyses were blinded to clinical outcomes. The analysis was repeated twice in random clinical samples (by D.E.V.) and the results were consistent and reproducible. A second investigator also confirmed the reproducibility of results using random clinical samples (J.G.Q). Experimental results were highly reproducible among several series of experiments and by different investigators (D.F-R, M,C-S, A.G-E, A.S-C, D.E-V., G.LR.).
Randomization	Randomization did not apply to this study since we did not test any specific therapy that required randomization. The study was designed as a prospective cohort study.
Blinding	Experimental and clinical imaging and atrial signal analyses were done by investigators blinded to clinical outcomes (D.E-V, J.G.Q, A.G-E). Proteomics, blood biomarkers, Western blots, histopathology and immunohistochemistry analyses were also done by investigators blinded to the experimental groups. Single cell computational simulations were done knowing the experimental changes in calcium handling proteins, which enabled us testing experimental conditions.

Reporting for specific materials, systems and methods

We require information from authors about some types of materials, experimental systems and methods used in many studies. Here, indicate whether each material, system or method listed is relevant to your study. If you are not sure if a list item applies to your research, read the appropriate section before selecting a response.

Materials & experimental systems

n/a	Involved in the study	
☐	☒	Antibodies
☒	☐	Eukaryotic cell lines
☒	☐	Palaeontology and archaeology
☐	☒	Animals and other organisms
☒	☐	Clinical data
☒	☐	Dual use research of concern
☒	☐	Plants

Methods

n/a	Involved in the study	
☒	☐	ChIP-seq
☒	☐	Flow cytometry
☒	☐	MRI-based neuroimaging

Antibodies

Antibodies used	1. ACTC1 Catalog number: 66125-1-Ig; Supplier: ProteinTech®<br>2. BAX Catalog number: M00183-1; Supplier: Boster Bio<br>3. MYBPC3 Catalog number: Sc-137237; Supplier: Santa Cruz Biotechnology<br>4. NCX Catalog number: MA3-926; Supplier: Thermo Fisher<br>5. PLN Catalog number: A010-14; Supplier: Badrilla<br>6. pThr17 PLN Catalog number: A010-13; Supplier: Badrilla<br>7. RyR2 Catalog number: MA3-916; Supplier: Thermo Fisher<br>8. pSer2814 RyR2 Catalog number: A010-31; Supplier: Badrilla<br>9. SERCA2 Catalog number: Sc-376235; Supplier: Santa Cruz Biotechnology<br>10. Polyclonal Goat Anti-Mouse (Ig/HRP) Catalog number: P044701-2; Supplier: Dako<br>11. Goat anti-rabbit (Ig/HRP) Catalog number: P044801-2; Supplier: Dako
Validation	1. ACTC1 Protein sequence Percent identity (Human vs pig): P68032 vs NP_001163988.1. 100.00%<br>2. BAX Protein sequence Percent identity (Human vs pig): Q07812.1 vs XP_003127338.2. 95.83%<br>3. MYBPC3 1 Protein sequence Percent identity (Human vs pig): Q14896 vs F1SID7. 81.00%<br>4. NCX Protein sequence Percent identity (Human vs pig): P32418 vs XP_020943964.1. 98.05%<br>5. PLN Protein sequence Percent identity (Human vs pig): P26678 vs NP_999378.1. 96.15%<br>6. pThr17 PLN 1 Protein sequence Percent identity (Human vs pig): P26678 vs NP_999378.1. 96.15%<br>7. RyR2 Protein sequence Percent identity (Human vs pig): Q92736 vs A0A5G2R1W3. 95.00%<br>8. pSer2814 RyR2 Protein sequence Percent identity (Human vs pig): Q92736 vs A0A5G2R1W3. 95.00%<br>9. SERCA2 1:1000 Protein sequence Percent identity (Human vs pig): P16615 vs NP_999030.1. 99.04%

Animals and other research organisms

Policy information about studies involving animals; ARRIVE guidelines recommended for reporting animal research, and Sex and Gender in Research

Laboratory animals	We used slow growing crossbred pigs (Yucatan-Large white) to minimize problems arising from handling over-large animals after long periods with AF. Males 60%. The average animal weight at the beginning of the study 54 Kg, average age 6 months.<br>- Justification of the use of pigs: The gold-standard model for translational research in cardiovascular disease is the pig (Lab Anim (NY). 2016;45:67-74. JACC Basic Transl Sci. 2020;5:840-856), which we use extensively at CNIC due to its anatomical and physiological similarities to humans. The pig heart´s size, morphology and electrophysiological properties are similar to the human heart, which makes it an ideal model to use in the study of atrial fibrillation. The adult atrial myocyte is a terminally differentiated cell and no continuous cell lines exist which replicate the electrical properties of the native atrial myocyte, and while mathematical models are useful in making predictions, they require validation and understanding of the human biology before they can be useful. Thus, there is currently no alternative to large animal experimentation when attempting to understand the consequences of long-term persistent atrial fbrillation.
Wild animals	No wild animals were used in this study.
Reporting on sex	Results should apply to both sexes. Both sexes were considered in the experimental design to reproduce clinical scenarios, with higher prevalence of atrial fibrillation in males than in females. We did not perform specific subanalysis of sex differences because of limited animals for sex specific subanalysis.
Field-collected samples	No field-collected samples were used in this study.
Ethics oversight	All animal procedures were approved by the Comunidad de Madrid (Ref# PROEX097/17 & PROEX078.8/21) and conformed to EU Directive 2010/63EU and Recommendation 2007/526/EC regarding the protection of animals used for experimental and other scientific purposes, enforced in Spanish law under Real Decreto 1201/2005.

Note that full information on the approval of the study protocol must also be provided in the manuscript.
